# Understanding virtual patients efficiently and rigorously by combining machine learning with dynamical modelling

**DOI:** 10.1007/s10928-021-09798-1

**Published:** 2022-01-05

**Authors:** Tongli Zhang, John J. Tyson

**Affiliations:** 1 Department of Pharmacology & Systems Physiology, College of Medicine, University of Cincinnati, 231 Albert Sabin Way, Cincinnati, OH 45219 USA; 2 Department of Biological Sciences, Virginia Polytechnic Institute & State University, Blacksburg, VA 24061 USA

**Keywords:** Quantitative systems pharmacology, Virtual patients, Machine learning, Bifurcation analysis, Nonlinear dynamics, Hypothalamic–pituitary–adrenal axis

## Abstract

Individual biological organisms are characterized by daunting heterogeneity, which precludes describing or understanding populations of ‘patients’ with a single mathematical model. Recently, the field of quantitative systems pharmacology (QSP) has adopted the notion of *virtual patients* (VPs) to cope with this challenge. A typical population of VPs represents the behavior of a heterogeneous patient population with a distribution of parameter values over a mathematical model of fixed structure. Though this notion of VPs is a powerful tool to describe patients’ heterogeneity, the analysis and understanding of these VPs present new challenges to systems pharmacologists. Here, using a model of the hypothalamic–pituitary–adrenal axis, we show that an integrated pipeline that combines machine learning (ML) and bifurcation analysis can be used to effectively and efficiently analyse the behaviors observed in populations of VPs. Compared with local sensitivity analyses, ML allows us to capture and analyse the contributions of simultaneous changes of multiple model parameters. Following up with bifurcation analysis, we are able to provide rigorous mechanistic insight regarding the influences of ML-identified parameters on the dynamical system’s behaviors. In this work, we illustrate the utility of this pipeline and suggest that its wider adoption will facilitate the use of VPs in the practice of systems pharmacology.

**Supplementary Information:**

The online version contains supplementary material available at 10.1007/s10928-021-09798-1.

## Introduction

The fields of molecular systems biology and systems pharmacology have achieved huge success by viewing living organisms as complex dynamical systems and utilizing mechanistic models to study how systems-level behaviors emerge from the interactions within underlying molecular control systems. With the expanding experience of mathematical modelers, employing powerful computational software and advanced algorithms, the complexity of biological control systems need no longer stop us from properly understanding how they function [1–4].

Because different patients with the same disease often respond differently to identical treatments, the field of quantitative systems pharmacology (QSP) has gone beyond the presumption of ‘one disease, one model’ and introduced that idea of *virtual patients* (VPs) to deal with the challenge of heterogeneity. Typically, VPs comprise a collection of mathematical models that have similar or identical structures (equations) and different parameter values, which are estimated from clinical data or selected from assumed probability distributions [5–9]. By mimicking a heterogeneous patient population and the different responses to identical treatments, the study of VPs helps to resolve the uncertainty deriving from patient heterogeneity during the medical decision-making process. The closer VPs are to real ones, the better they can be used to understand and design clinical trials.

The increased utilization of VPs also brings new computational challenges. For example, traditional sensitivity analysis has been used to investigate the dependence of model dynamics on changing parameter values. In local sensitivity analysis, the change in some output of a model (say, the steady state of a dynamical variable) is measured in response to a small change in a single parameter. In global sensitivity analysis, multiple parameters are changed simultaneously and the resulting changes in model behavior are analysed [10, 11]. It is straightforward to carry out sensitivity analysis for a single model with a fixed set of parameter values; however, since each VP in a cohort has a different set of parameter values, must we carry out a new sensitivity analysis for each VP? This would demand a tremendous amount of computational and human resources when tens of thousands of VPs need to be examined.

Furthermore, how can we associate the physiological behaviors exhibited by a population of VPs to underlying differences in molecular control circuits? Parameter changes necessarily result in different behaviors of a mathematical model, but how can these changes in system-level behaviors be associated with the specific parameter changes? In addition to quantitative behavior changes associated with specific changes in one or more parameters, can we also explore the qualitative potential of a dynamical model in dependence on general movements through parameter space?

To cope with these practical challenges, we propose an integrated pipeline that combines *machine learning* (ML) with *bifurcation analysis*. ML is able to efficiently explore the behaviors of thousands of VPs and quickly discover the parameters that are most significant in determining some specific response of these VPs [12, 13]. In this work we show how to supplement ML with bifurcation analysis, in order to provide deeper, mechanistic insights into how the qualitative dynamics of the mathematical model (i.e., the systems-level behavior of the model) emerge from changes of specific parameter values. Our study suggests that a wider adoption of this pipeline may contribute to model-informed drug development in the future by providing efficient and rigorous analysis of VPs as representatives of pharmacological treatments in clinical practice.

## Methods

### Ordinary differential equations for the model

The four differential equations governing time-dependent changes in corticotropin-releasing hormone (CRH), adrenocorticotropic hormone (ACTH), cortisol (COR) and glucocorticoid receptor (GR) are all of ‘standard form’:

1 $$\frac{d{X}_{i}}{dt}= {k}_{i} \left({F}_{i}-{X}_{i}\right),$$

2 $${\text{for which}}, {F}_{i}=\frac{1}{1+{e}^{-\sigma {W}_{i}}},$$

3 $${\text{and }} W_{i}= {R}_{0}^{i} + \sum_{j}{R}_{j}^{i}\cdot {X}_{j}.$$

The ‘indices’ *i* and *j* are assigned to the names of the model variables, which are *CRH*, *ACTH*, *COR* and *GR*. The ODE file for the simulations and the parameter values used in our simulations are given in Supplementary Tables 1 and 2. A more detailed description of the approach can be found in the literature [14, 15] as well as in our previous publications [16] and [17].

The stress signal always starts from a low level, *Stress* = 0.1. At *t* = 10, *Stress* begins to increase exponentially, $Stress=0.1{e}^{0.6(t-10)}$. For *t* > 15, *Stress* decreases exponentially at the same rate, $Stress=2{e}^{0.6(t-15)}$ (Supplementary Fig. 1).

### Generation of virtual patients and virtual populations

All time series simulations were carried out with the free software XPPaut (http://www.math.pitt.edu/~bard/xpp/xpp.html) using the stiff algorithm for temporal simulations. *VPs* were generated by randomly changing the parameters in the ODE model; parameter values were chosen from uniform distributions over the ranges reported in Supplementary Table 2. The computer simulations of all VPs were analysed and plotted with MATLAB (https://www.mathworks.com/).

For each VP, the COR level was first allowed to reach steady state (SS before), then a transient stress signal was applied, and the VP was allowed to reach a new steady state after the stress signal disappeared (SS after). By comparing the SS levels before and after, we assigned each VP to its appropriate ‘population’: higher VPs have SS after > SS before, lower VPs have SS after < SS before, and control VPs have SS after = SS before. We generated 1000 VPs for each of the three categories (3000 VPs in total). Early exploratory computations indicated that 1000 VPs per category are sufficient for ML analysis to deliver robust and consistent results. The parameter distribution of these VPs are illustrated in Supplementary Fig. 2.

### Machine learning analysis

Two separate ML analyses were carried out: one comparing higher VPs to control VPs, and the other comparing lower VPs to control VPs. Both Random Forest analysis and Decision Tree (DT) analysis were carried out in R (https://www.r-project.org/) [18], using the parameter values of VPs as input and their identities (higher, lower, or control) as classifiers. Classification Trees were computed using the rpart2 algorithm in R. With recursive partitioning, the algorithm finds optimal threshold values of parameters that can be used to classify the VPs most efficiently. Using these thresholds, the VPs in a mother node were then split into two daughter nodes.

Random Forest analysis (the caret package in R) was used to assemble multiple DTs, to avoid overfitting and to compute a rank for each parameter’s contribution/importance to prediction accuracy. Default settings were used along with 10-fold cross-validation. Parameter contribution was calculated as the reduction in accuracy resulting from permuting the predictors. The computed importances were then normalized so that the maximal feature importance is 100 and the minimal value is 0.

Support Vector Machine (SVM) identifies hyperplanes that classify different types of virtual populations. Since we used only two significant parameters (${R}_{0}^{CRH} {\text{and}} {R}_{CRH}^{CRH}$ when distinguishing higher VPs from control VPs; and ${R}_{0}^{ACTH}$ and ${R}_{0}^{CRH}$ when distinguishing lower VPs from controls) for this classification, the SVM boundaries were identified as curves in two-dimensional planes. The binary classification through SVM was carried out with the *fitcsvm* function of MATLAB (https://www.mathworks.com/).

Readers who are interested in additional technical details on these ML methods are encouraged to contact the corresponding author.

### Bifurcation analysis

For bifurcation analysis, representative VPs, whose parameter values are reported in Supplementary Table 2, were selected. For the one-parameter bifurcation analysis, *Stress* is held constant as a parameter. First, the system is allowed to reach its steady state at a low value of *Stress*; then *Stress* is gradually increased, and the steady state response of the system (both stable and unstable steady states) are computed as functions of *Stress*.

Loci of stable and of unstable steady states in a one-parameter bifurcation diagram coalesce at characteristic values of *Stress*, called saddle-node bifurcation points. For values of *Stress* between these saddle-node points, the systems is bistable [19]. In a two-parameter bifurcation diagram, these saddle-node bifurcation points are followed in dependence on *Stress* and some other model parameter deemed to be ‘important’ by ML analysis.

Both one- and two-parameter bifurcation analyses were carried out with the free software Oscill8 (http://oscill8.sourceforge.net). After the diagrams were computed, they were plotted with MATLAB (https://www.mathworks.com/).

## Results

### Multiple interacting feedback loops contribute to heterogeneity of virtual patients

**Fig. 1:**
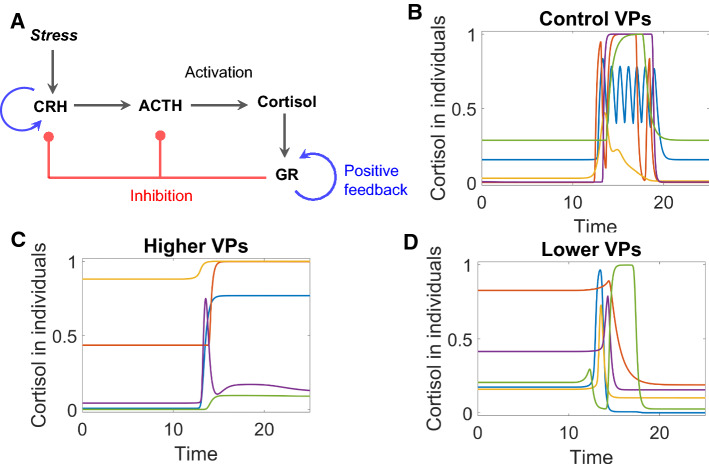
**A** Interaction diagram of the HPA model. *CRH* corticotropin-releasing hormone, *ACTH* adrenocorticotropic hormone, *GR* glucocorticoid receptor. Black arrows denote activation; blue arrows, positive feedback; red links, negative feedback. **B–D** Typical time-course simulations (using a different color for each simulation) of control VPs (virtual patients), higher VPs (virtual patient whose cortisol levels are higher after stress than before), and lower VPs (virtual patients whose cortisol levels are lower after stress)

The hypothalamic–pituitary–adrenal (HPA) axis (Fig. 1A) plays a significant role in stress response and psychiatric diseases [20–23]. Stress signals induce a sequential release of CRH, ACTH, and then COR. COR then influences target cells through the GR. The HPA axis is characterized by negative feedback, by which the release of CRH and ACTH are repressed once GR is activated [23–25]. The HPA axis also includes two positive feedbacks on GR [26] and CRH [27]. Our mathematical model of the HPA axis is described in the ‘Methods’ section.

Simulations of the model with random combinations of parameter values resulted in three populations of VPs with divergent behaviors in response to stress. In all three populations, COR level increases transiently in response to a brief pulse of stress. In ‘control’ VPs, COR level then falls to the same level as before stress (Fig. 1B); in ‘higher’ VPs, COR level after stress is sustained higher than before stress (Fig. 1C); in ‘lower’ VPs, COR level after stress is lower than before (Fig. 1D). The simulated VPs mimic real patients, who are characterized by heterogeneous COR responses to stress [22, 28–30]. We consider control VPs to represent the normal physiological response of the HPA axis, and the higher and lower VPs to represent various degrees of ‘pathological’ responses.

In this way, our VPs exhibit heterogeneous dynamical behaviors due presumably to complex regulation of the HPA axis by the interactions of multiple feedback loops. How to map such heterogeneity to the underlying regulatory system is a general challenge when we try to understand the responses of VPs, and we illustrate how to cope with this challenge in the following paragraphs.

### Machine learning identifies the most significant parameters, ${{\varvec{R}}}_{0}^{{\varvec{C}}{\varvec{R}}{\varvec{H}}}\;\mathbf{a}\mathbf{n}\mathbf{d }\;{{\varvec{R}}}_{{\varvec{C}}{\varvec{R}}{\varvec{H}}}^{{\varvec{C}}{\varvec{R}}{\varvec{H}}}$, in distinguishing higher VPs from control VPs

To analyze the simulation data using a ML approach, we assembled the parameter values and identifier (higher, lower or control) for each VP of the population into the rows of a matrix. ML analysis uses the parameter values as input features and the population identity as output. The parameters in our mathematical model (see ‘Methods’ section) are of the form: ${R}_{j}^{i}$ = influence of species *j* on the pseudo-steady state value of species *i*, and ${k}_{i}$ = rate constant for approach of species *i* to its pseudo-steady state value. Parameters of the form ${R}_{0}^{i}$ determine the pseudo-steady state of species *i* when all variables ${X}_{j}=0.$ The indices *i* and *j* are assigned the ‘values’ *CRH*, *ACTH*, *COR* and *GR*, in accordance with Fig. 1A.

**Fig. 2:**
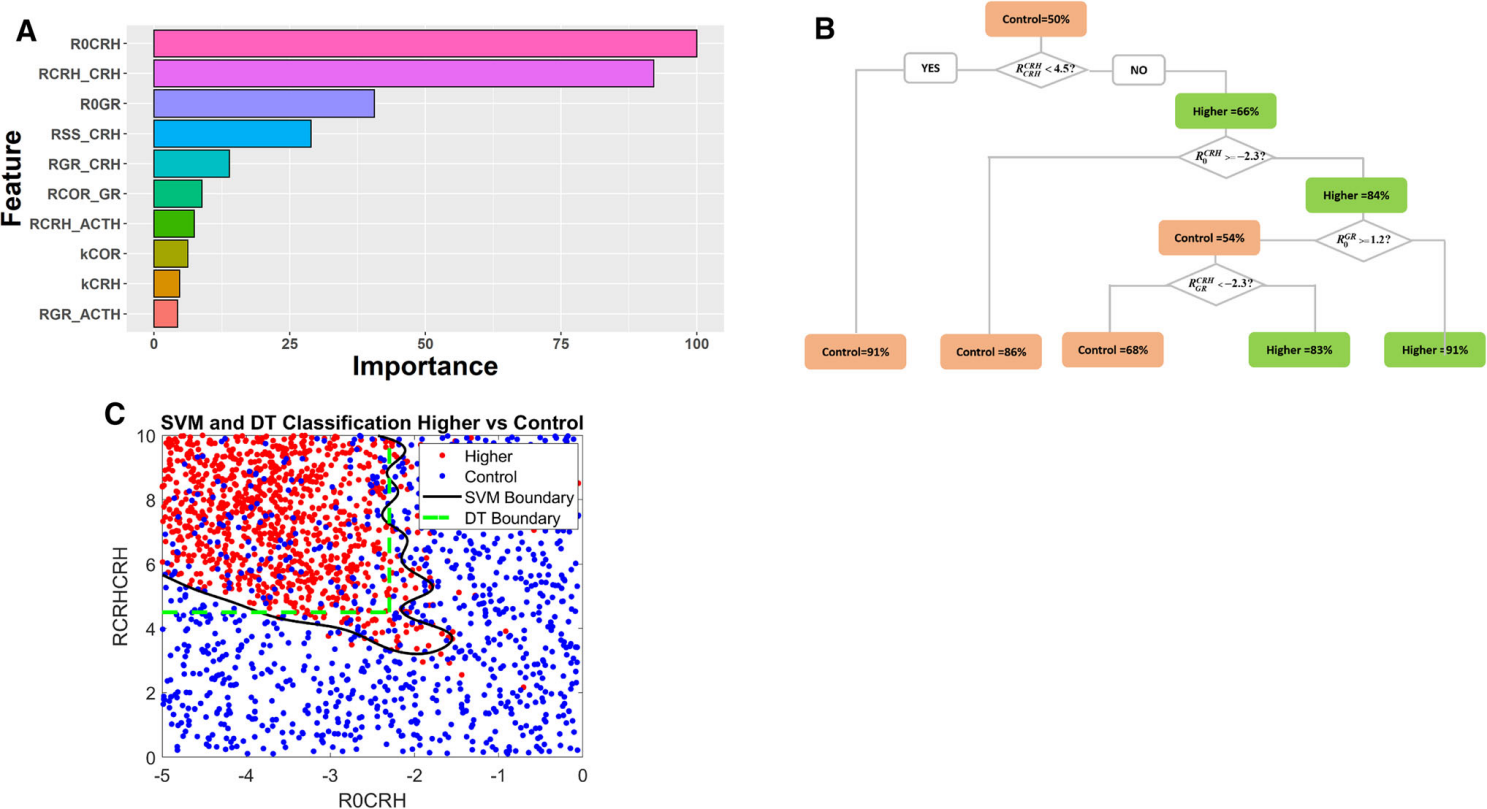
**A** Parameter ranking by Random Forest analysis. ${R}_{0}^{CRH} {\text{ and }} {R}_{CRH}^{CRH}$ are the most important parameters that distinguish higher VPs from control VPs. **B**. Decision Tree analysis of higher VPs and control VPs. The percentage of dominant VPs (whose percentage ≥ 50%), either control or higher VPs, is indicated in each node. Orange nodes indicate that the percentage of control VPs ≥ higher VPs; green nodes, otherwise. Starting from the top, VPs in a mother node are split into two daughter nodes based on comparing the value of a control parameter to a threshold value. All VPs meeting the splitting criteria are sorted to the left node, while those not meeting the criteria go to the right node. **C**. Support Vector Machine (SVM) analysis cross validates the results of Decision Tree (DT) analysis. Red and blue dots represent the higher- and control VPs, while the black and green curves sketch out the boundaries identified by SVM analysis and DT analysis (panel **B**)

The ‘control’ VPs serve as the control group for ML. Adopting a strategy of ‘divide and conquer’, we first compared the higher VPs with the control group and later the lower VPs with controls. One thousand control VPs and one thousand higher VPs were subjected to Random Forest analysis, which could distinguish higher VPs from control VPs with high accuracy (Supplementary Table 2). Random Forest’s feature-importance analysis (parameter-contribution) indicated that the parameters ${R}_{0}^{CRH}$, ${R}_{CRH}^{CRH}$ and ${R}_{0}^{GR}$ play the most important roles in distinguishing between the two populations (Fig. 2A).

In order to cross validate the results of Random Forest analysis, the same patient data and input features were subjected to DT analysis. Indeed, the key parameter features identified by Random Forest play significant roles in the DT to distinguish higher VPs from controls (Fig. 2B). In the root node of the tree (top node), there are equal numbers of control VPs and higher VPs (50% each). Most of the VPs with ${R}_{CRH}^{CRH}<4.5$ are *control* (the orange node at the bottom left, 91% control VPs). On the other hand, if ${R}_{CRH}^{CRH}\ge 4.5$, then 66% of the VPs are *higher* (green node, upper right). A better classification of higher VPs can be achieved by requiring that ${R}_{0}^{CRH}<-2.3$ and ${R}_{0}^{GR}<-1.2$, in which case 91% of the VPs (green node at bottom right) were characterized by higher COR levels after stress.

A further cross validation was carried out with SVM analysis (Fig. 2C). In order to illustrate these results, we performed SVM analysis with the same VP data and two key components (${R}_{CRH}^{CRH}$ and ${R}_{0}^{CRH}$) as input features. Though SVM and DT drew different boundaries in the two-dimensional plane defined by ${R}_{CRH}^{CRH}$ and ${R}_{0}^{CRH}$ (Supplementary Table 2), both methods reached the consistent conclusion that the majority of VPs at the top left (${R}_{CRH}^{CRH}\ge 4.5$ and ${R}_{0}^{CRH}<-2.3$) are characterized by higher COR levels after stress, while the majority of VPs outside this region are controls.

Cross-validation of these results with three different ML methods boosted our confidence that the important roles attributed to ${R}_{CRH}^{CRH}$ and ${R}_{0}^{CRH}$ are unlikely due to algorithm or computational bias, but more likely to be consequences of some hidden characteristics of the VPs.

**Fig. 3:**
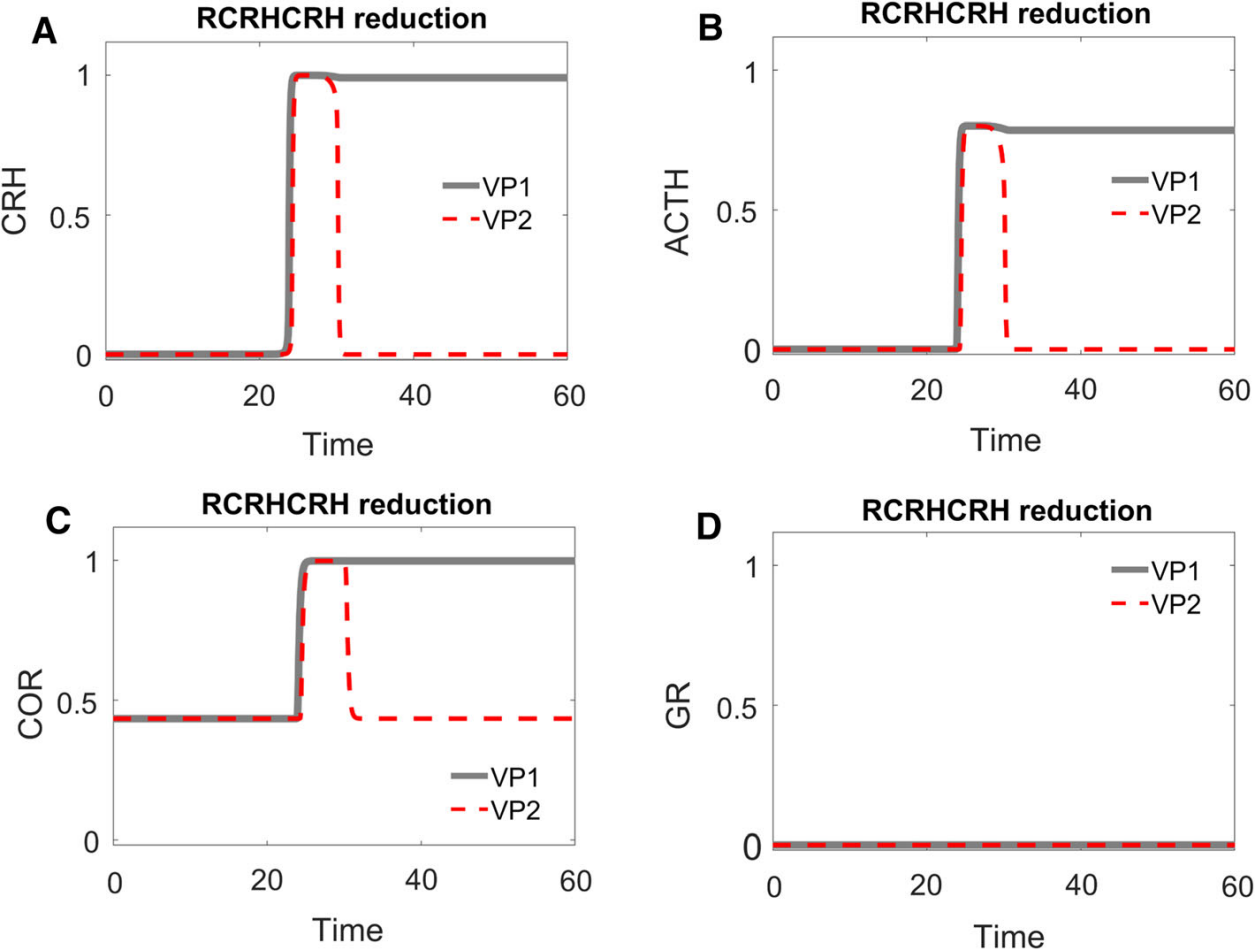
Time-course simulations of two VPs: VP1 (grey solid curves) has higher cortisol level after transient stress, while VP2 (red dashed curves) is identical to VP1 except for a lower value of ${R}_{CRH}^{CRH}$. Notice that the level of CRH (**A**), low before stress, is elevated by the stress and then either remains high in VP1 or drops in VP2 after the stress signal disappears. ACTH (**B**) follows the trend of CRH, due to its activation by CRH, and cortisol (COR, **C**) follows the pattern of ACTH. GR (**D**), on the other hand, remains low during the whole time in both VPs; it does not get activated to any great extent by the stress stimulation. The parameter values for all VPs are provided in Supplementary Table 2

Following the clues provided by ML analysis, we randomly chose a sample VP (VP1) with higher COR level after stress and generated a second VP (VP2) by reducing its value of ${R}_{CRH}^{CRH}$. The time dependent trajectories of VP1 and VP2 were shown as grey solid curves and red dashed curves in Fig. 3, respectively. In VP1 (grey solid curves in Fig. 3A–D), the level of CRH increased and then stayed high after the stress was relieved, which resulted in a higher level of ACTH and COR after stress. On the other hand, the level of GR remained low throughout the stress response. In VP2 (red dashed curves in Fig. 3A–D), CRH started inactive and COR was moderate. Upon the elevation of the stress signal, CRH and ACTH were activated and COR was released. The release of COR was only transient, though. After the stress signal returns to normal, the level of COR drops back to its original level. Hence, a simple decrease of the value of ${R}_{CRH}^{CRH}$ can move a VP from the higher population (VP1) to the control population (VP2). The simulation results were consistent to the significant role of ${R}_{CRH}^{CRH}$ identified by ML in distinguishing the population of higher VPs from the population of control ones.

### One-parameter bifurcation analysis explains the higher (pathological) cortisol level after transient stress stimulation

Though the ML analysis efficiently identifies the parameters that most effectively distinguish higher VPs from control VPs, it does not explain how these particular parameters account for the responses of higher VPs. To investigate this question, we proceeded with a refined dynamical analysis of these two ‘sample’ VPs, VP1 and VP2.

**Fig. 4:**
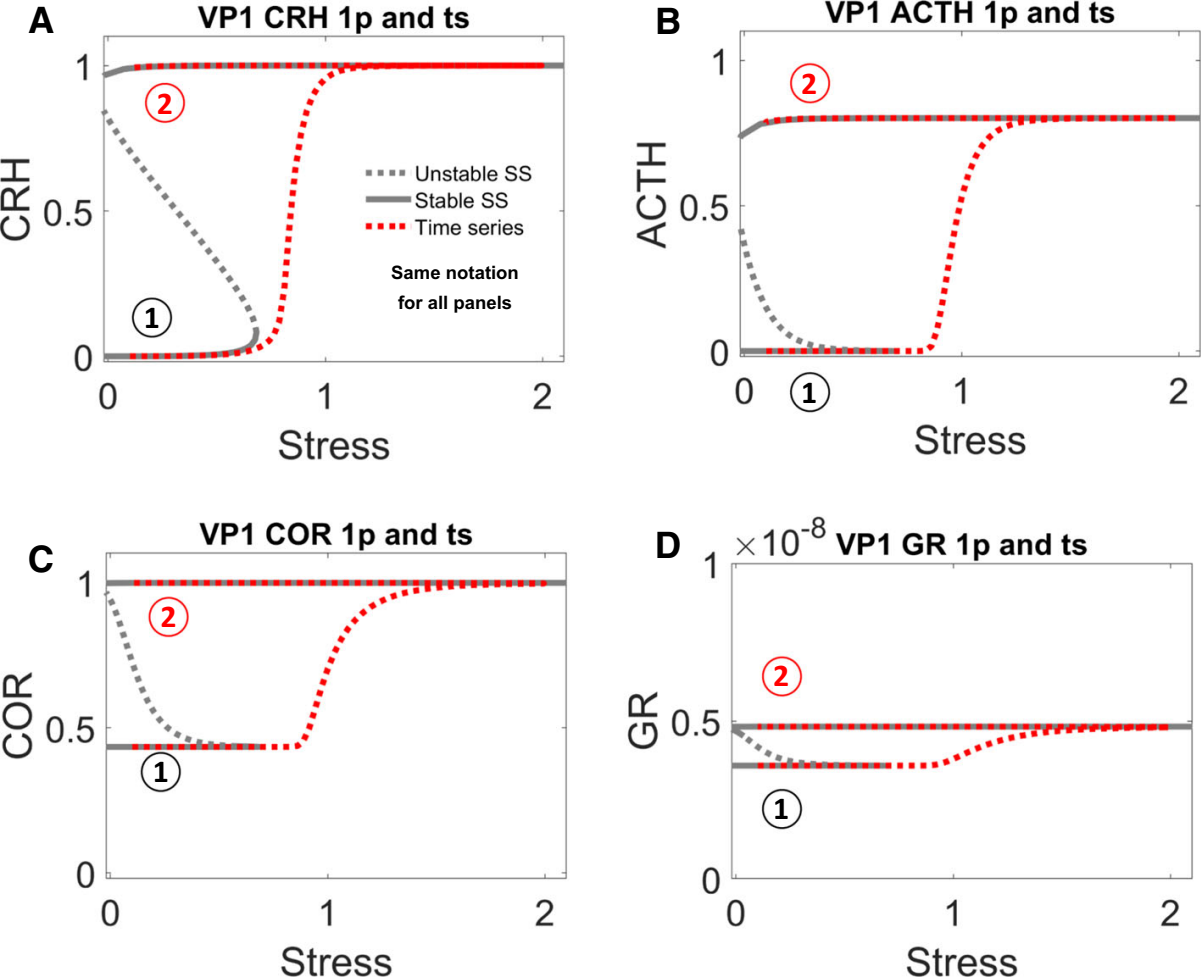
**A–D** One-parameter bifurcation diagrams for CRH, ACTH, COR and GR, respectively. In these bifurcation diagrams, grey solid curves indicate stable steady states, grey dashed curves indicate unstable steady states, and red dotted curves trace time-course simulations (see Fig. 3) across the relevant bifurcation diagrams. Position 1 indicates the system’s state before stress begins; position 2 after stress ends. In panels **A–C**, positions 1 and 2 are characterized by the same low level of stress before and after the transient stimulus, but a higher level of response after the stress disappears. Panel **D** (note the change in scale of the vertical axis) indicates that GR remains ‘inactive’ during the whole process

The sustained activation of CRH is due to its positive feedback, which is not counteracted by negative feedback from GR (Fig. 1A) in cases when GR remains low throughout the stress response. With appropriate parameter settings, the positive feedback on CRH results in a bistable switch, indicated by the S-shaped curve of CRH steady state (Fig. 4A) as a function of stress level. The lower and upper branches of the S-shaped curve correspond to stable steady states (‘nodes’) of CRH abundance, and the middle branch (dashed) corresponds to unstable steady states (‘saddle points’). For *Stress* < 0.7, CRH level can be either low or high. For *Stress* > 0.7, the HPA control system settles on a unique steady state of CRH level. At *Stress* = 0.7, the unstable steady state of intermediate CRH level and the stable steady state of low CRH level coalesce and disappear at a ‘saddle-node’ bifurcation point, leaving only the stable steady state of high CRH.

For an unstressed patient (*Stress* = 0.1), we assume that CRH resides on the lower branch (position 1 in Fig. 4A). Following a sufficient elevation of stress (*Stress* > 0.7, in this case), CRH is activated (i.e., brought to the upper branch of the switch), as illustrated by the red trajectory in Fig. 4A. The stress is temporary, and, as it drops, CRH level stays on the upper branch of the bistable switch (position 2 in Fig. 4A). As a consequence of this bistability, positions 1 and 2 had identical stress level (*Stress* = 0.1), but their CRH response levels are dramatically different.

Although ACTH is not subject to positive feedback itself, it also exhibits bistable response due to its activation by CRH. Before stress is applied, inactive CRH results in a low level of ACTH (position 1 of Fig. 4B); after transient stress stimulation, the sustained activation of CRH sustains a high activity of ACTH (position 2 of Fig. 4B). In the same manner, COR also inherits a bistable response due to activation of ACTH (Fig. 4C). The level of GR, however, remains low, both before and after stress stimulation, because the inactivation rate of GR (${R}_{0}^{GR}<-1.2$) is too strong to be overcome by the released COR (Fig. 4D). With a lower rate of inactivation (i.e., ${R}_{0}^{GR}$ less negative), GR would be activated, as discussed later.

### One-parameter bifurcation diagram reveals how the reduction of ${{\varvec{R}}}_{{\varvec{C}}{\varvec{R}}{\varvec{H}}}^{{\varvec{C}}{\varvec{R}}{\varvec{H}}}$ alters the dynamical behaviors of the VPs

As discussed above, the bistable activation of COR by the stress signal is essential for the higher VPs to sustain higher COR levels after transient stress stimulation. Bearing this in mind, we proceeded to examine how such bistable activation is regulated by the significant parameters identified by ML analysis.

**Fig. 5:**
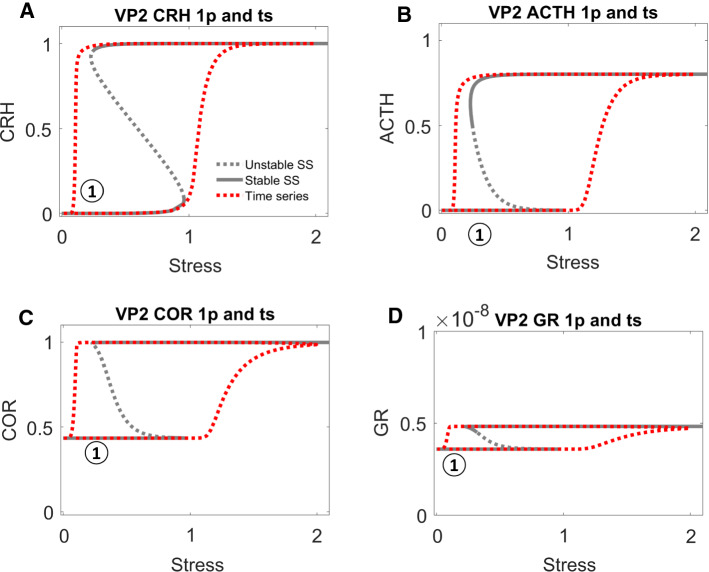
One-parameter bifurcation analysis for the ‘control’ VP2 in Fig. 3, which was derived from ‘higher’ VP1 by reducing the value of ${R}_{CRH}^{CRH}$

For this purpose, we again computed the one-parameter bifurcation diagram with *Stress*, with the value of ${R}_{CRH}^{CRH}$ reduced to that in VP2 (Fig. 5). The red trajectory of VP2 in Fig. 5 was taken from VP2, whose dynamics were plotted in Fig. 4. Due to its lower value of ${R}_{CRH}^{CRH}$, VP2 no longer resides in the bistable region at normal stress level (*Stress* = 0.1). Its COR level increased transiently upon a transient stress stimulation. Then, after the stress signal decreased to normal, its COR level returned to normal level.

Comparing Figs. 4 and 5, we can extract the take home message: a pathological attractor (i.e., the stable steady state labelled by a red 2 in Fig. 4) is responsible for the pathological COR level observed in higher VPs, e.g., patients with post-traumatic stress disorder (PTSD). If this pathological attractor is removed (as in Fig. 5), the patient can be cured.

### Machine learning identifies ${{\varvec{R}}}_{0}^{{\varvec{A}}{\varvec{C}}{\varvec{T}}{\varvec{H}}}$ as the most significant parameter in distinguishing lower VPs from control VPs

**Fig. 6:**
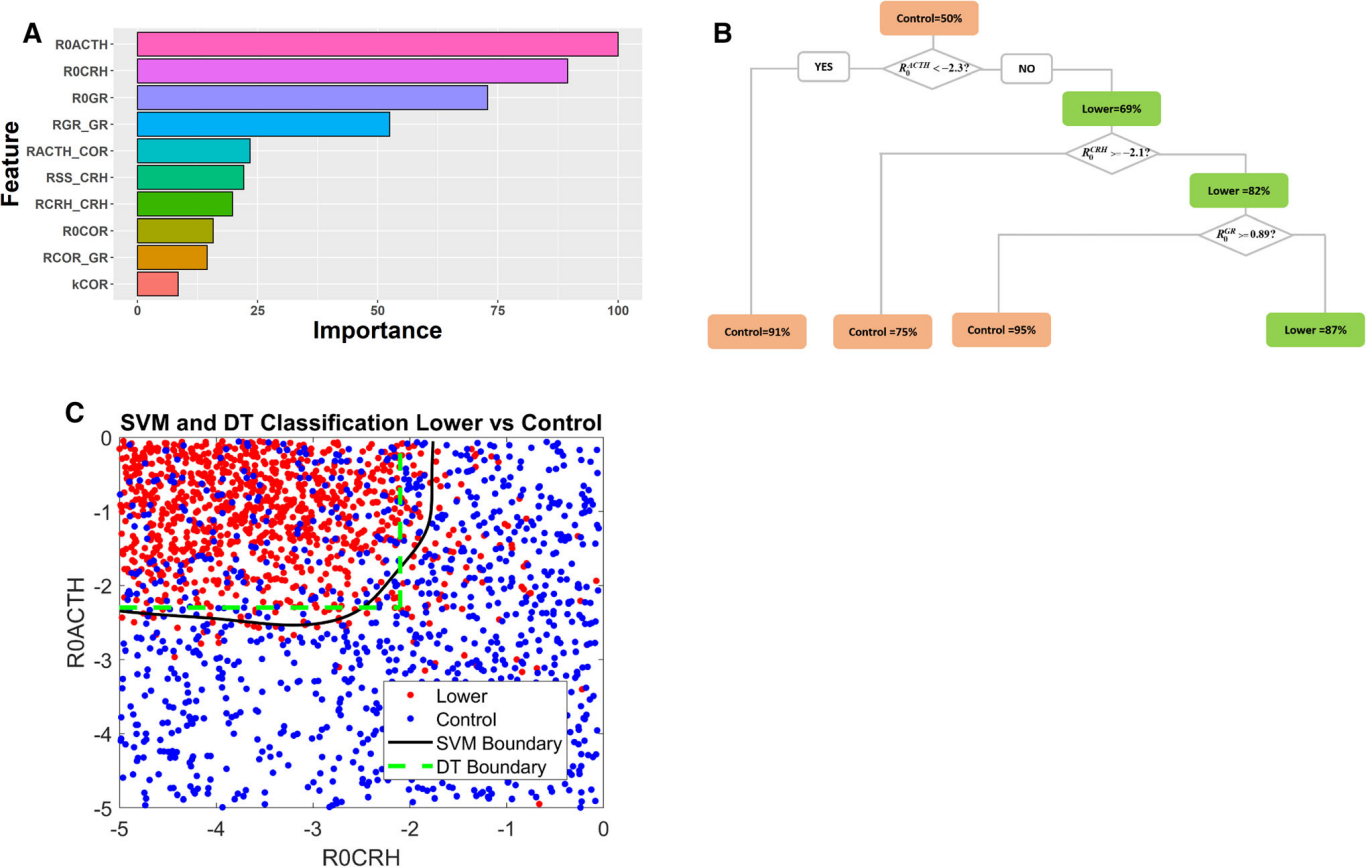
Machine learning analysis of lower VPs compared to control VPs. For notations, refer to legend of Fig. 2. **A** Parameter ranking by Random Forest analysis. ${R}_{0}^{ACTH}$ is the most important parameter in distinguishing these two populations. **B** Decision Tree analysis of higher VPs and control VPs, which showed that 91% of VPs with ${R}_{0}^{ACTH}<-2.3$ are control VPs. **C** Support Vector Machine analysis and Decision Tree cross-validated each other. SVM and DT drew similar boundaries in this two-dimensional plane and consistently concluded that ${R}_{0}^{ACTH}$ and ${R}_{0}^{CRH}$ are the key parameters distinguishing lower VPs from control ones

We next carried out an ML comparison of populations of lower VPs and control VPs. For cross validation purpose, several ML methods were utilized. The input features and performance of these methods are summarized in Supplementary Table 3. Random Forest analysis identified ${R}_{0}^{ACTH} {\text{ and }} {R}_{0}^{CRH}$ as the most important parameters in distinguishing these two populations (Fig. 6A). This result was further supported by DT analysis and SVM analysis, which showed that if ${R}_{0}^{ACTH}$ is smaller than a critical threshold (− 2.3), most of the VPs meeting this criteria are control VPs (bottom left orange node in Fig. 6B, C).

**Fig. 7:**
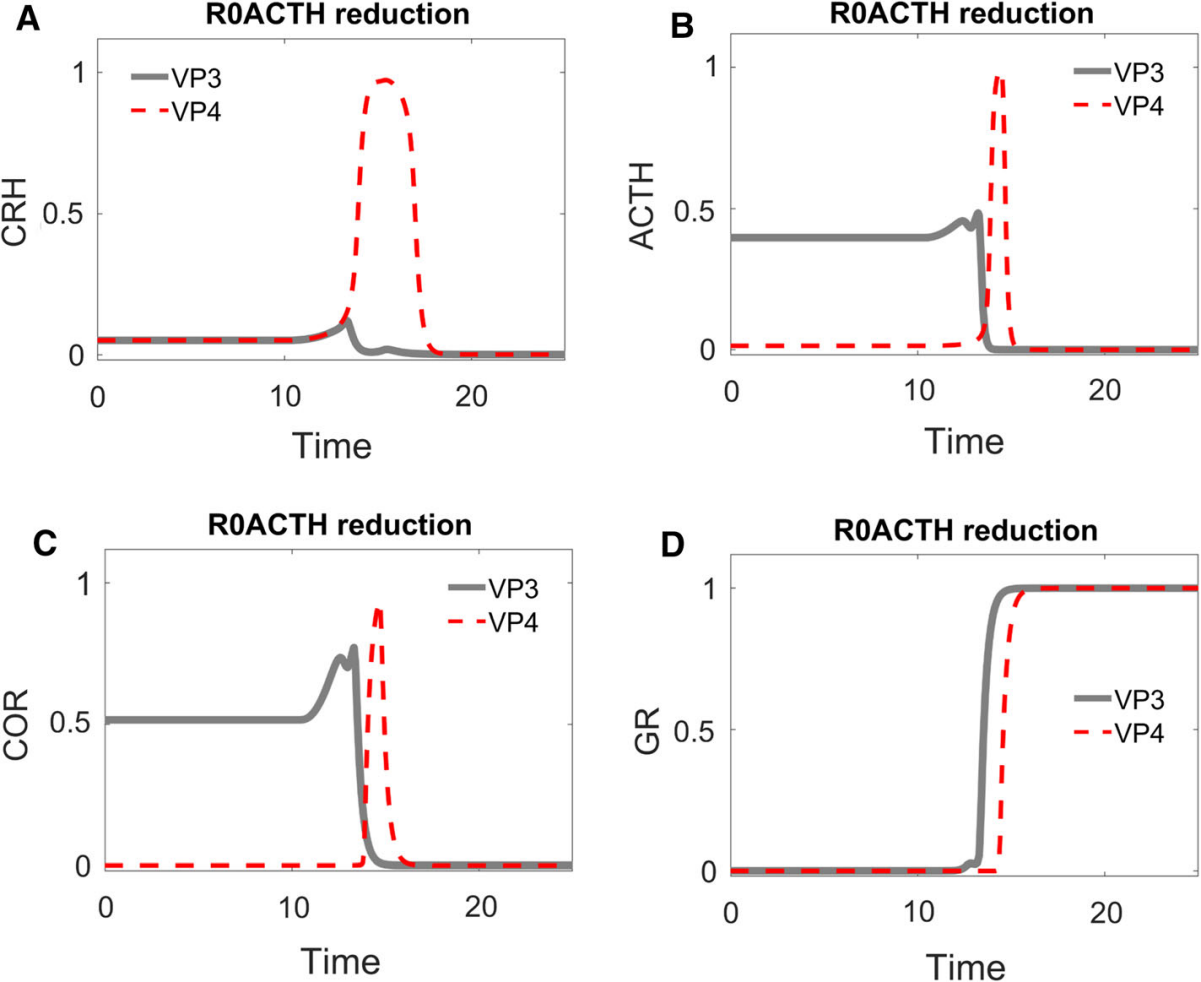
Time-course simulations of two distinct VPs: VP3 (grey solid curves) is a sample VP with lower cortisol level after stress; VP4 (red dashed curves) is identical to VP3 except for a lower value of ${R}_{0}^{ACTH}$. The time-dependent changes of CRH (**A**), ACTH (**B**), cortisol (**C**), and GR (**D**) are illustrated. See text for elaboration of the dynamics of VP3 and VP4

We then double checked the role of ${R}_{0}^{ACTH}$ with time series simulations (Fig. 7). For this purpose, we chose a sample (VP3) from the population of lower VPs and reduced its ${R}_{0}^{ACTH}$ value (while keeping other parameter values unchanged) to create VP4. The simulation of VP3 (grey solid curves in Fig. 7) behaves as categorized: starting with an intermediate value of COR, its COR level elevates temporarily upon stimulation by the stress signal, then the level of COR decreases and stays at a lower level compared with that before stress. Consistent with the significant role of ${R}_{0}^{ACTH}$, the reduction of ${R}_{0}^{ACTH}$ alone makes VP4 behave as a control VP. After the transient stress stimulation, VP4’s COR level returns to the same level that it had before stress (red dashed curves in Fig. 7). Reduction of ${R}_{0}^{ACTH}$ changes the dynamics of CRH, ACTH, and COR; meanwhile, the time-dependent trajectory of GR is barely altered by the change (Fig. 7).

The ‘cure’ exhibited by VP4 (Fig. 7, red dashed curves) is spurious: VP4 is ‘control’ (SS after = SS before) because COR level ‘before’ is very low to start with! This case can hardly be considered a cure. If low COR level caused the symptoms in VP3, these symptoms would not disappear in VP4; indeed, VP4 would experience those symptoms in the resting, unstressed state. So, reduction of ${R}_{0}^{ACTH}$ does not result in a satisfactory cure. Next, we turned to bifurcation analysis to investigate why reduction of ${R}_{0}^{ACTH}$ does not cure VP3.

### Bifurcation analysis reveals the systems-level properties of lower VPs

**Fig. 8:**
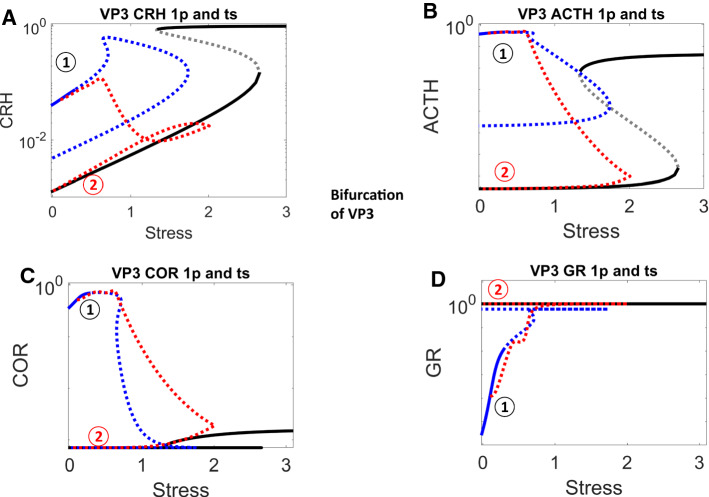
One-parameter bifurcation analysis for the ‘lower’ VP3 in Fig. 7. The four panels show the bifurcation diagram for CRH, ACTH, COR and GR. For notation see the legend to Fig. 4. In panels **A–D**, positions 1 and 2 are characterized by the same low level of stress before and after the transient stimulus, but a lower level of CRH (**A**), ACTH (**B**), and cortisol (**C**), and a higher level of GR (**D**)

We next carried out bifurcation analysis (Fig. 8) for the sample lower VP3, whose time series simulations were shown as the grey curves in Fig. 7 and replotted as the red dotted curves in Fig. 8. This VP started with intermediate levels of CRH, ACTH and COR before stress stimulation (position 1 in Fig. 8A–C), and low GR activity (position 1 in Fig. 8D).

Stress stimulation results in elevation of all four components (Fig. 8A–D). After the stress signal returns to normal, GR activity is sustained high by its positive feedback (position 2 in Fig. 8D). The sustained activation of GR then inactivates CRH and ACTH through the negative feedback (position 2 in Fig. 8A, B), which consequently results in the inactivation of COR (position 2 in Fig. 8C).

**Fig. 9:**
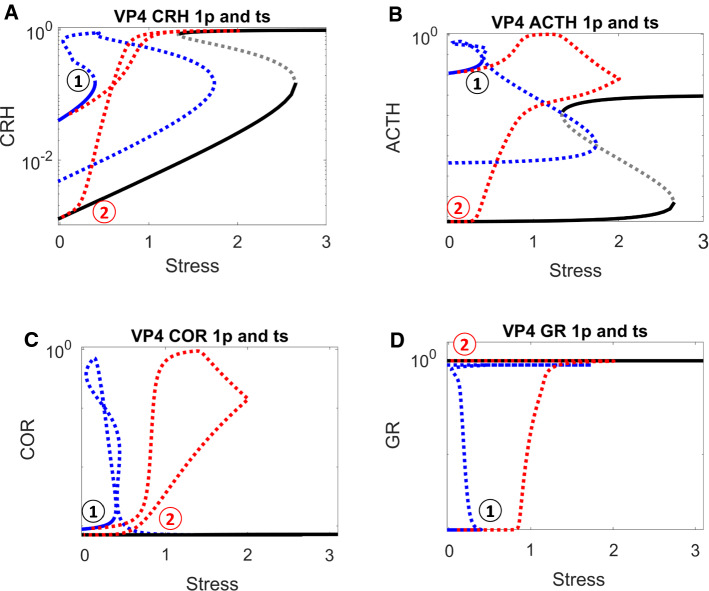
One-parameter bifurcation analysis for the ‘control’ VP4 in Fig. 7, which was derived from ‘lower’ VP3 by reducing the value of ${R}_{0}^{ACTH}$. In panels **A–D**, positions 1 and 2 are characterized by the same low level of stress, but a lower level of CRH (**A**) and ACTH (**B**), and a higher level of GR (**D**); the level of cortisol (**C**) is very low both before and after stress

A reduction of ${R}_{0}^{ACTH}$ alters these bifurcation diagrams (Fig. 9): less significantly for the CRH and GR diagrams, more significantly for the ACTH and COR diagrams. The altered bifurcations diagrams explain the altered time series dynamics in VP4 with reduced ${R}_{0}^{ACTH}$ (red curves in Figs. 7, 9). Starting from a low level, GR is activated by the stress signal and sustained by its positive feedback (Fig. 9D). Through the negative feedbacks, activated GR then represses CRH and reduces its level (Fig. 9A). On the other hand, the reduced value of ${R}_{0}^{ACTH}$ results in a low level of ACTH even before stress stimulation. ACTH is transiently activated by the stress signal, then it returns to an even lower level after stress is relieved (Fig. 9B). However, since such low levels of ACTH are insufficient to activate COR, COR levels both before and after the stress stimulation are low and do not show detectable difference (Figs. 7C, 9C).

In this way, bifurcation analysis reveals that for VPs to have lower pathological COR levels after stress stimulation, their physiological COR levels must be sufficiently high before stress. This insight generally holds for multiple VPs (Supplementary Fig. 3). In these individuals, high GR activity sustained by its positive feedback can then reduce the COR level through its negative feedback effects. This analysis leads to a testable prediction. Individual patients who are characterized by higher COR levels under physiological conditions before stress should be more likely to experience lower (pathological) levels of COR after stress. On the other hand, individuals whose COR levels are low under physiological conditions should be less likely to develop lower-COR pathology post stress.

What is more, the bifurcation analysis revealed the reason why reduction of ${R}_{0}^{ACTH}$ could not cure VP3. Though VP4 has reduced level of ${R}_{0}^{ACTH}$ and its bifurcation diagram is quantitively altered, the qualitative features of its bifurcation diagram remained unchanged. VP4, like VP3, still has the pathological attractor (labelled as red 2 in Figs. 8, 9). Consequently, both VP3 and VP4 are stuck in this pathological attractor and would experience symptoms that would result from low COR level.

### Bifurcation analysis reveals that a reduction in the level of ${{\varvec{R}}}_{{\varvec{G}}{\varvec{R}}}^{{\varvec{G}}{\varvec{R}}}$ could remove the pathological attractor

**Fig. 10:**
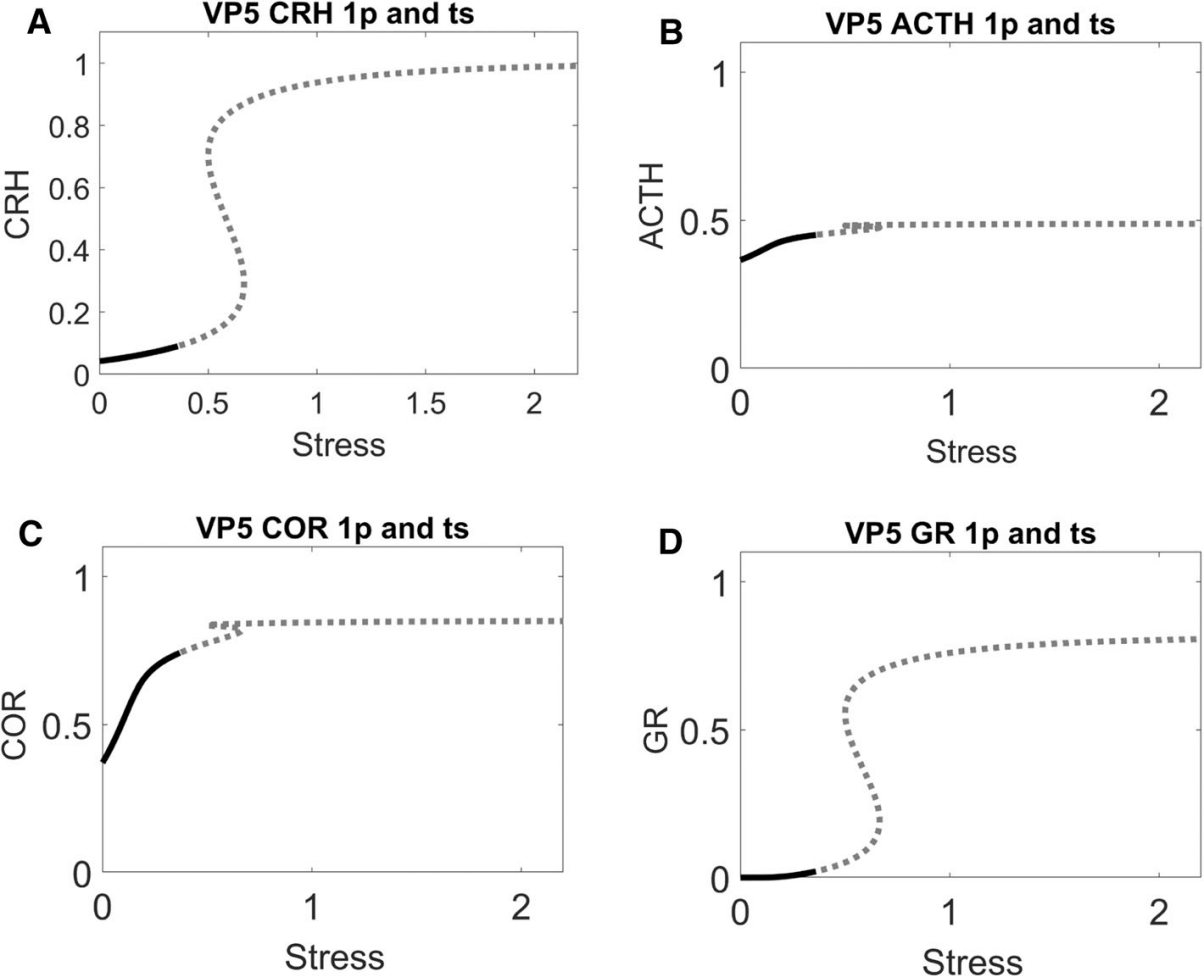
One-parameter bifurcation analysis for a newly designed ‘control’ VP5, which was derived from ‘lower’ VP3 by reducing the value of ${R}_{GR}^{GR}$. The stress-dependent changes in steady state levels of CRH (**A**), ACTH (**B**), cortisol (**C**) and GR (**D**) are plotted. At low stress, each component is characterized by a stable steady state (black solid curve). At higher levels of stress (above ~ 0.4), the steady state of each component becomes unstable (indicated by grey dashed curves)

In order to design a better cure, we then carried out bifurcation analysis with the other control parameters. Bifurcation analysis suggests that a reduction of ${R}_{GR}^{GR}$ would be able to remove the pathological attractor (Fig. 10): starting from a stable steady state, CRH, ACTH, COR and GR enter an oscillatory region when stress increases (indicated by dashed curves in Fig. 10). Importantly, only one stable attractor exists when stress is at its low, resting level.

**Fig. 11:**
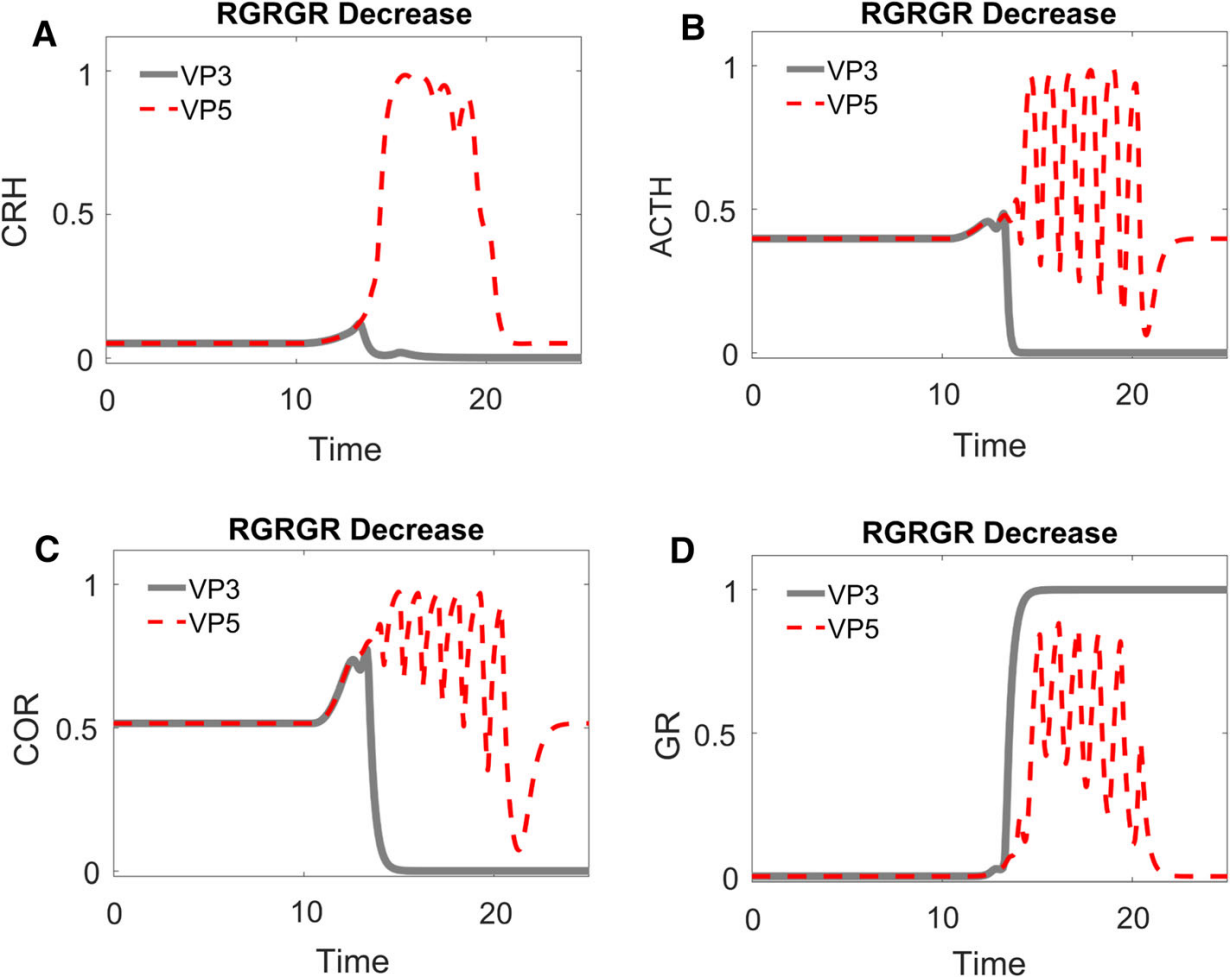
Time-course simulations of two distinct VPs: VP3 (grey solid curves) is the same sample VP used in Fig. 7; VP5 (red dashed curves) is identical to VP3 except for a lower value of ${R}_{GR}^{GR}$. The time-dependent changes of CRH (**A**), ACTH (**B**), cortisol (**C**), and GR (**D**) are illustrated. Note how the cortisol level of VP5 starts at a moderate value (0 < *t* < 10), shows transient oscillations as stress builds up (10 < *t* < 15), and then returns to its initial value after stress is relieved (*t* > 20)

Time series simulation confirmed that the reduction of ${R}_{GR}^{GR}$ resulted in a more satisfactory COR response to stress. Transient increase of stress results in oscillation of all four components (VP5, Fig. 11A–D). After the stress signal returns to normal, all four components returned to their normal, resting value. Hence though VP5 could still suffer from transient symptoms associated with fluctuating COR, the symptoms would eventually disappear after COR returns to its normal state.

## Discussion

**Fig. 12:**
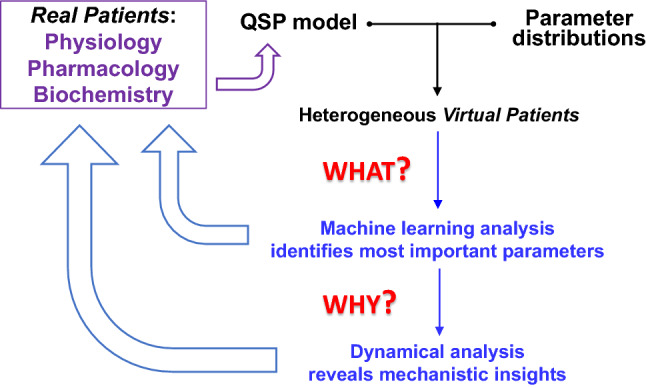
How our computational workflow fits into the current practice of virtual patient analysis. Data from real patients (physiology, pharmacology and biochemistry) suggests a quantitative systems-pharmacology (QSP) model, such as the proposed mechanism of the HPA axis in Fig. 1A and its mathematical formulation in ‘Methods’ section. By subjecting the mathematical model to random adjustments of its parameters, we create a population of *virtual patients*. Applying machine learning algorithms to this population, we quickly identify the most important parameters governing some relevant response of the VPs (i.e., *what’s* in charge?). This information can be fed back to the data, to see if the influential features of the model, identified by ML, correspond to influential features in the real-patient data. For a better understanding of patient responses, we address the *why* question by dynamical systems theory. Bifurcation theory can provide significant insights into the mechanisms behind the most important features. These insights offer further feedback to the real-patient data. To the extent that this workflow is successful, we make progress in treating real diseases. If the features identified by this workflow do not correspond well with patient experiences, the discrepancies will suggest ways to modify the QSP model in order to improve the predictions of ML and bifurcation analyses

Currently, there is great interest in combining mechanistic modelling and ML, as evidenced by the literature [31–37] and by this special issue. In this work, we have proposed a computational workflow for the efficient and robust analysis of heterogeneous populations of VPs (Fig. 12) and provided a *proof of principle* example based on stress responses of the HPA axis. By a combination of ML and dynamical analysis, this computational pipeline can reveal rigorously computed, interpretable insights into the behavior of VPs governed by a complex molecular regulatory network. Such insights will not only help model developers to calibrate and validate VP populations but also help model users (who are not necessarily model developers themselves) to gain a deeper understanding of both the advantages and limitations of complex models, to modify the models to meet their own purposes, and to properly utilize the models in making critical decisions. We believe that this approach will promote the development of new, effective tools for systems pharmacology [33].

### Our workflow

Our workflow takes advantage of the distinct strengths of ML and mechanistic modelling, allowing each method’s strengths to overcome the other’s limitations. ML is a ‘black-box’ method that quickly identifies features of significant impact and provides targets for a refined analysis by dynamical systems theory. On the other hand, bifurcation analysis is an ‘open-box’ method that reveals how underlying molecular mechanisms account for the features identified by ML. Together, these two methods provide timely and understandable analysis of the heterogeneity of VPs.

However, it would be a mistake to think of ML algorithms or dynamical systems models as ‘genies’ that magically transform data streams into reliable suggestions for pharmaceutical treatments of some human pathology. Rather, these analytical methods help us to think more clearly about the molecular underpinnings of cell and organismal physiology and to trace more rigorously the consequences of interfering with these mechanisms pharmacologically [38]. For instance, as our example (the HPA axis) illustrates, machine-learning classifications suggest sensible interventions for restoring ‘higher’ VPs to normality, but for ‘lower’ VPs, they direct us to completely unreasonable treatments that put the ‘patient’ in a pathological state before as well as after the stress. Dynamical models, on the other hand, might avoid such pitfalls, but they are stymied by the sheer immensity of the parameter space that must be sampled in searching for reasonable interventions. (For example, to solve the HPA model for all integer values of the parameters over the ranges specified in Supplementary Table 2, we would have to do 3 × 10^14^ simulations, which would take 10 years at 10^6^ simulations per second.) But the two methods can be usefully combined by supplementing the rapid-but-superficial correlations identified by ML algorithms with the deep mechanistic insights provided by dynamical models. (For example, we have shown here how one-parameter bifurcation diagrams can reveal how pharmaceutically inducible changes of specific kinetic parameters of a control system may alter its dynamical behavior in beneficial directions.) The broad and deep understanding of complex QSP models afforded by our computational workflow has the potential to contribute significantly to drug development, provided these models are rigorously validated and verified [39].

### The hypothalamic–pituitary–adrenal axis

The example described here—the HPA axis—indicates that complex QSP models can be effectively understood with a combination of ML and bifurcation analysis. Although the HPA axis may seem simple, both its structure (the interaction of multiple positive and negative feedback loops) and its dynamical behaviors (combined bistable switches and oscillations) are complex enough to illustrate the power of our analytical workflow.

Nonlinear feedbacks—both positive and negative—potentially result in complex behaviors (switches and oscillators) within distinct regions of parameter space, and these complex dynamical behaviors may—or may not—be observed in the corresponding physiological context [40, 41]. Our workflow identifies the parameter regions where these behaviors occur. If the behaviors are observed, as in the case of heterogeneous COR levels in patients with stress disorders [12, 18–20], our approach can provide strong constraints on the parameter values that should be used to construct representative VPs. On the other hand, if the behaviors are not observed, then model developers can use this knowledge to avoid parameter regions that would construct unacceptable VPs.

### Post-traumatic stress disorder

We chose to illustrate our workflow with the HPA axis because it is intimately involved in PTSD, a sustained pathological response to an acute stressful experience. Patients experiencing PTSD can be classified into low-COR and high-COR cohorts [42, 43], and there is considerable discussion among clinicians as to the significance of these differences [44–46]. Our analysis of how the HPA axis may respond to stress can help to clarify the origins of PTSD and suggest practical interventions in the clinic. First of all, our mathematical analysis of the HPA axis makes clear that pathologically high or low COR levels after acute stress are related to heterogeneities among individual patients in the dynamical parameters that characterize the stress response. Some individuals are resistant to PTSD, some are prone to low-COR PTSD, and some to high-COR PTSD. ML methods can identify the most important parameters associated with each of these three responses in populations of VPs. Most importantly, dynamical systems analysis of these VP populations points compellingly to ‘bistability’ of the HPA response as the origin of pathological COR responses in PTSD. A dynamical system is bistable if it may persist in two different, stable steady states of response for the same level of an input signal; e.g., a low level of stress may be consistent with either a normal or a pathological level of COR in the blood stream. An acute and sufficiently high level of stress may push an individual from the normal state into the pathological state, and the patient remains in the stable, pathological state even after the stress disappears.

Our simulation analysis has defined PTSD pathology as the difference between COR level before and after stress. With this practical definition, we have classified the *VPs* to be either of higher (COR increases after stress), lower (COR decreases after stress), or control (COR does not change upon transient stress stimulation).

Dynamical analysis with these VPs suggests that bistability underlies the pathological COR changes of PTSD patients. If this understanding of the origins of PTSD is true, it suggests three different possibilities for clinical intervention. First of all, our analysis suggests that the pathological state coexists with the normal state, but the patient is trapped by the local stability of the pathological state. In this case, it might be possible to kick the patient out of the pathological state and into the ‘domain of attraction’ of the normal state by a transient perturbation of the HPA axis, say, by COR injection (if COR level is low in the pathological state) or by inhibiting CRH (if the COR level is too high). Secondly, it should be possible to permanently treat PTSD patients by drugs that interfere with the ‘important’ parameters identified by ML analysis and whose modes of action are revealed by one-parameter bifurcation diagrams. For example, Fig. 2C suggests that high-COR PTSD could be treated by drugs that impair the positive feedback on CRH by decreasing ${R}_{CRH}^{CRH}$. For patients with low-COR PTSD, Fig. 6C suggests increasing ${R}_{0}^{CRH}$(making CRH more responsive). Alternatively, one might give preventative doses of appropriate drugs to persons prone to PTSD (e.g., soldiers) before they are sent into stressful circumstances. The drug cocktail used will depend, of course, on the person’s PTSD-class: high- or low-COR response after transient stress.

## Conclusion

Both ML methods and bifurcation analysis are applicable, in principle, to models of arbitrary complexity; hence, it is possible (again, in principle) to apply our workflow to models of commercial interest, with many more differential equations and parameters than our example of the HPA axis. In practice, however, there are limits to the complexity of systems that can be handled by present-day computational tools. Our workflow could be enhanced by combining it with model-simplification methods, such as time scale separation and modularity. In this way, we could approach a fuller understanding of realistic QSP models of human diseases.

Clearly, mathematical tools need continuous improvements to meet the ever-growing needs of biomedical and pharmaceutical industries. To deal with a variety of diseases in real patients with different genetic backgrounds and environments, treated by diverse sets of drugs, it will be necessary to improve and refine both our modelling framework and our analytical pipeline [12, 13, 47]. Assiduous improvements of the pipeline will provide the QSP community with powerful analytical tools to understand mechanisms, to make sense of data, and to guide our challenging endeavour to identify and optimize treatments for complex human diseases.

## Supplementary Information

Below is the link to the electronic supplementary material.

Supplementary file1 (DOCX 29 kb)

Supplementary file2 (PPTX 1461 kb)
