# Supplementary material for: Understanding virtual patients efficiently and rigorously by combining machine learning with dynamical modelling
Source: J Pharmacokinet Pharmacodyn. 2022 Jan 5;49(1):117–31. doi: 10.1007/s10928-021-09798-1 (PMC8837571; doi:10.1007/s10928-021-09798-1)
Supplement: Supplementary file 1 — Supplementary file1 (DOCX 29 kb) [file 10928_2021_9798_MOESM1_ESM.docx]

**Supplementary Table 1.** ODEs of the model.

F(Wi,sigma) = 1/(1+exp(-sigma*Wi))

W_CRH = R0CRH + RCRH_CRH*CRH + RSS_CRH*Stress + RGR_CRH*GR

W_ACTH= R0ACTH+ RCRH_ACTH*CRH + RGR_ACTH*GR

W_COR = R0COR + RACTH_COR*ACTH

W_GR = R0GR + RCOR_GR*COR + RGR_GR*GR

dCRH/dt = kCRH*(F(W_CRH,2)-CRH)

dACTH/dt = kACTH*(F(W_ACTH,2)-ACTH)

dCOR/dt = kCOR*(F(W_COR,2)-COR)

dGR/dt = kGR*(F(W_GR,5)-GR)

dStress/dt = kStr*Stress

Stress(0) = 0.1

# For 0 < t < 10, kStr=0.

# For 10 < t < 15, kStr=0.6.

# For t > 15, kStr=-0.6.

**Supplementary Table 2.** Parameter values for VPs, with differences highlighted. VP1~5 are the five virtual patients whose simulations and bifurcation analysis are shown in the main figures.

| Name | Range | | VP1 (higher) | VP2 (control) | VP3 (lower) | VP4 (control) | VP5 (control) |
| --- | --- | --- | --- | --- | --- | --- | --- |
| $R_{0}^{CRH}$ | | [-5,0] | -4.769 | -4.769 | -1.7246 | -1.7246 | -1.7246 |
| $R_{0}^{ACTH}$ | | [-5,0] | -4.6243 | -4.6243 | -0.5777 | -2.5 | -0.5777 |
| $R_{0}^{COR}$ | | [-5,0] | -0.13298 | -0.13298 | -3.6256 | -3.6256 | -3.6256 |
| $R_{0}^{GR}$ | | [-5,0] | -3.934 | -3.934 | -4.1604 | -4.1604 | -4.1604 |
| $R_{SS}^{CRH}$ | | [0,10] | 4.4088 | 4.4088 | 0.71403 | 0.71403 | 0.71403 |
| $R_{CRH}^{ACTH}$ | | [0,10] | 5.3218 | 5.3218 | 7.1935 | 7.1935 | 7.1935 |
| $R_{ACTH}^{COR}$ | | [0,10] | 4.1263 | 4.1263 | 9.2009 | 9.2009 | 9.2009 |
| $R_{COR}^{GR}$ | | [0,10] | 0.10454 | 0.10454 | 4.5697 | 4.5697 | 4.5697 |
| $R_{CRH}^{CRH}$ | | [0,10] | 6.7519 | 5 | 3.8288 | 3.8288 | 3.8288 |
| $R_{GR}^{GR}$ | | [0,10] | 5.1742 | 5.1742 | 7.131 | 7.131 | 0.7 |
| $R_{GR}^{CRH}$ | | [-10,0] | -7.0634 | -7.0634 | -1.6143 | -1.6143 | -1.6143 |
| $R_{GR}^{ACTH}$ | | [-10,0] | -8.1015 | -8.1015 | -8.16 | -8.16 | -8.16 |
| $k_{CRH}$ | | [0,10] | 8.3713 | 8.3713 | 3.9102 | 3.9102 | 3.9102 |
| $k_{ACTH}$ | | [0,10] | 9.6369 | 9.6369 | 8.0665 | 8.0665 | 8.0665 |
| $k_{COR}$ | | [0,10] | 4.3075 | 4.3075 | 3.3582 | 3.3582 | 3.3582 |
| $k_{GR}$ | | [0,10] | 5.4486 | 5.4486 | 3.3212 | 3.3212 | 3.3212 |

**Supplementary Table 3.** Model performance metrics

| Model | Virtual patient data set | Input features | Accuracy |
| --- | --- | --- | --- |
| Random Forest | 1000 higher VPs and 1000 control ones | All parameters | 92.7 % |
|  | 1000 lower VPs and 1000 control ones | All parameters | 93.1% |
| Decision Tree | 1000 higher VPs and 1000 control ones | All parameters | Labelled in nodes |
|  | 1000 lower VPs and 1000 control ones | All parameters | Labelled in nodes |
| Support Vector Machine | 1000 higher VPs and 1000 control ones | $R_{0}^{CRH} \text{and} R_{CRH}^{CRH}$ | 87% |
|  | 1000 lower VPs and 1000 control ones | $R_{0}^{ACTH}$and $R_{0}^{CRH}$ | 83% |

**Supplementary Figure legends**

**Supplementary Figure 1**. Time-course of the stress signal applied identically to all VPs.

**Supplementary Figure 2A-D.** Distributions of parameter values in higher, control and lower VPs.

**Supplementary Figure 3A-F.** One-parameter bifurcation analysis for additional ‘lower’ virtual patients (VPs). Although these bifurcation diagrams show quantitative differences, they are qualitatively similar to each other.
