# Supplementary material for: Understanding virtual patients efficiently and rigorously by combining machine learning with dynamical modelling
Source: J Pharmacokinet Pharmacodyn. 2022 Jan 5;49(1):117–31. doi: 10.1007/s10928-021-09798-1 (PMC8837571; doi:10.1007/s10928-021-09798-1)
Supplement: Supplementary file 2 — Supplementary file2 (PPTX 1461 kb) [file 10928_2021_9798_MOESM2_ESM.pptx]

## Slide 1
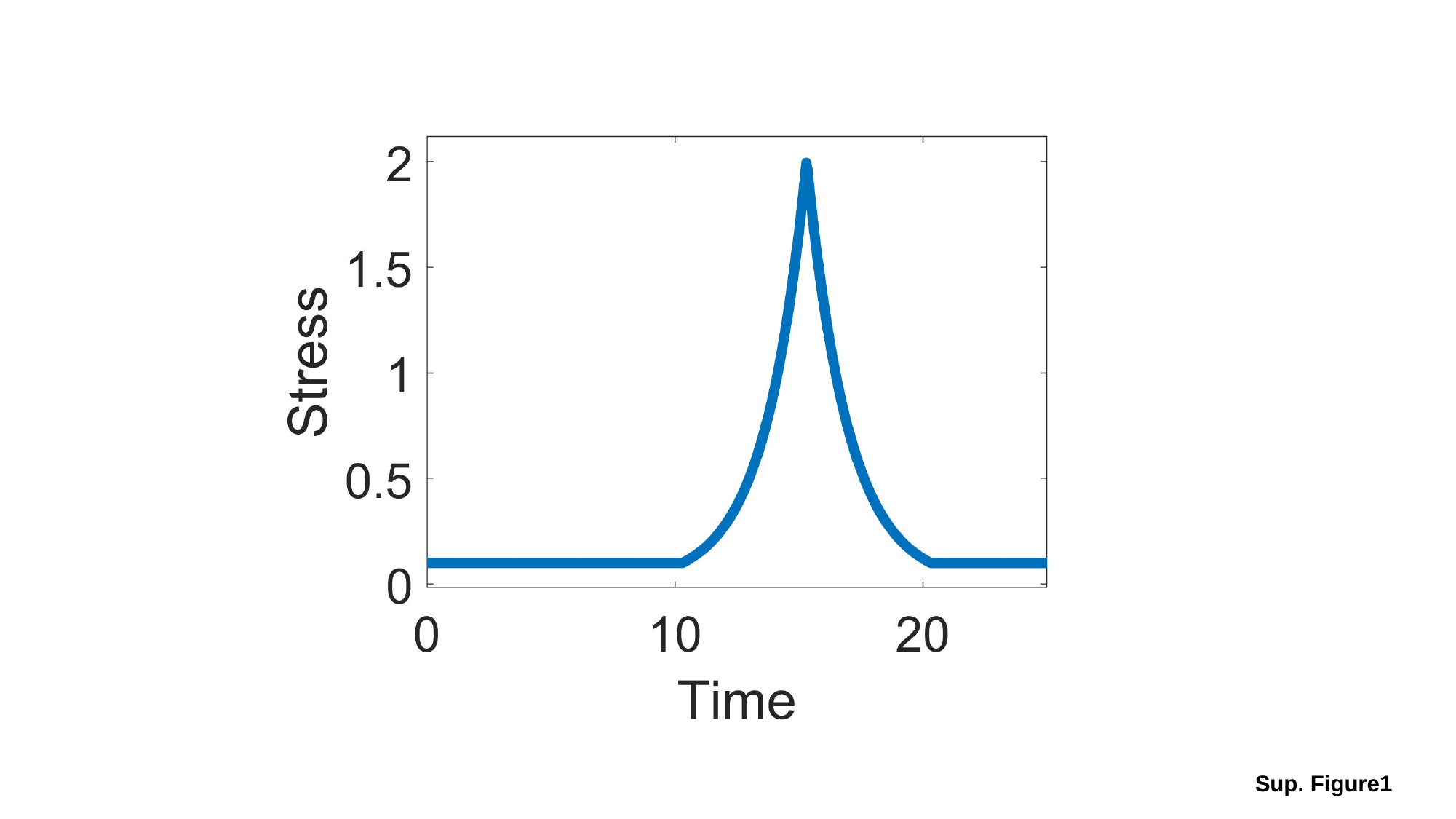

Sup. Figure1

## Slide 2
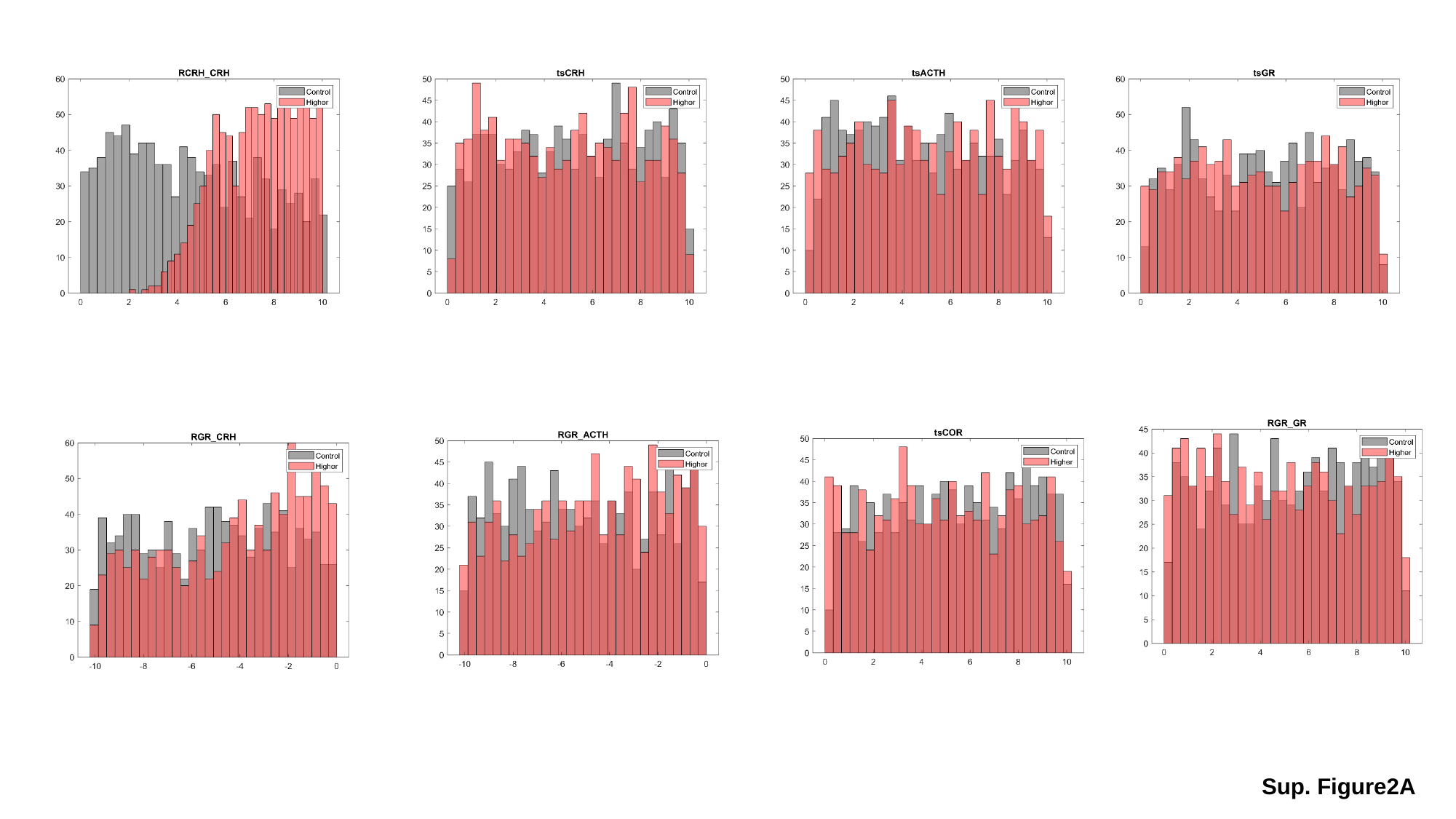

Sup. Figure2A

## Slide 3
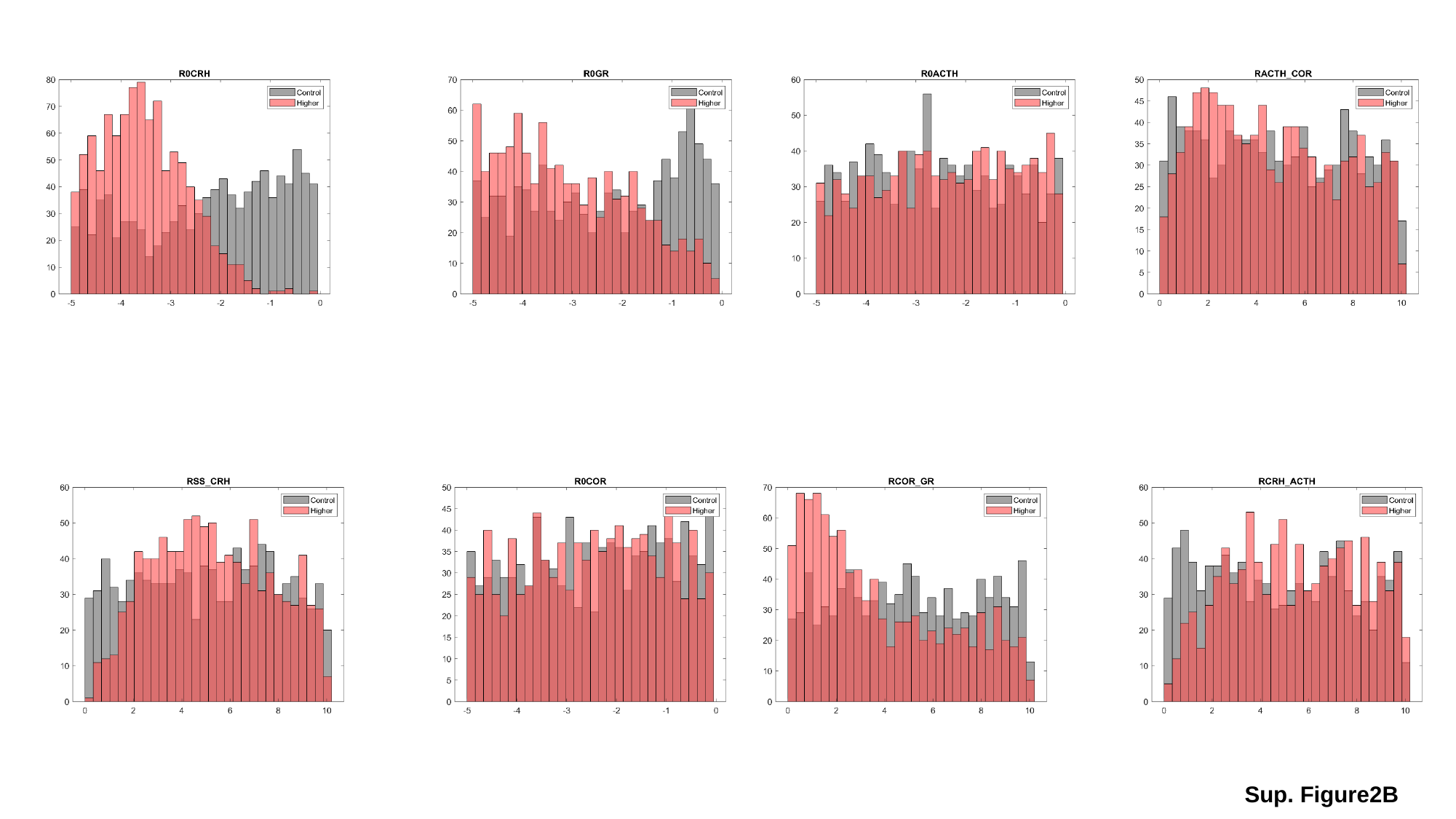

Sup. Figure2B

## Slide 4
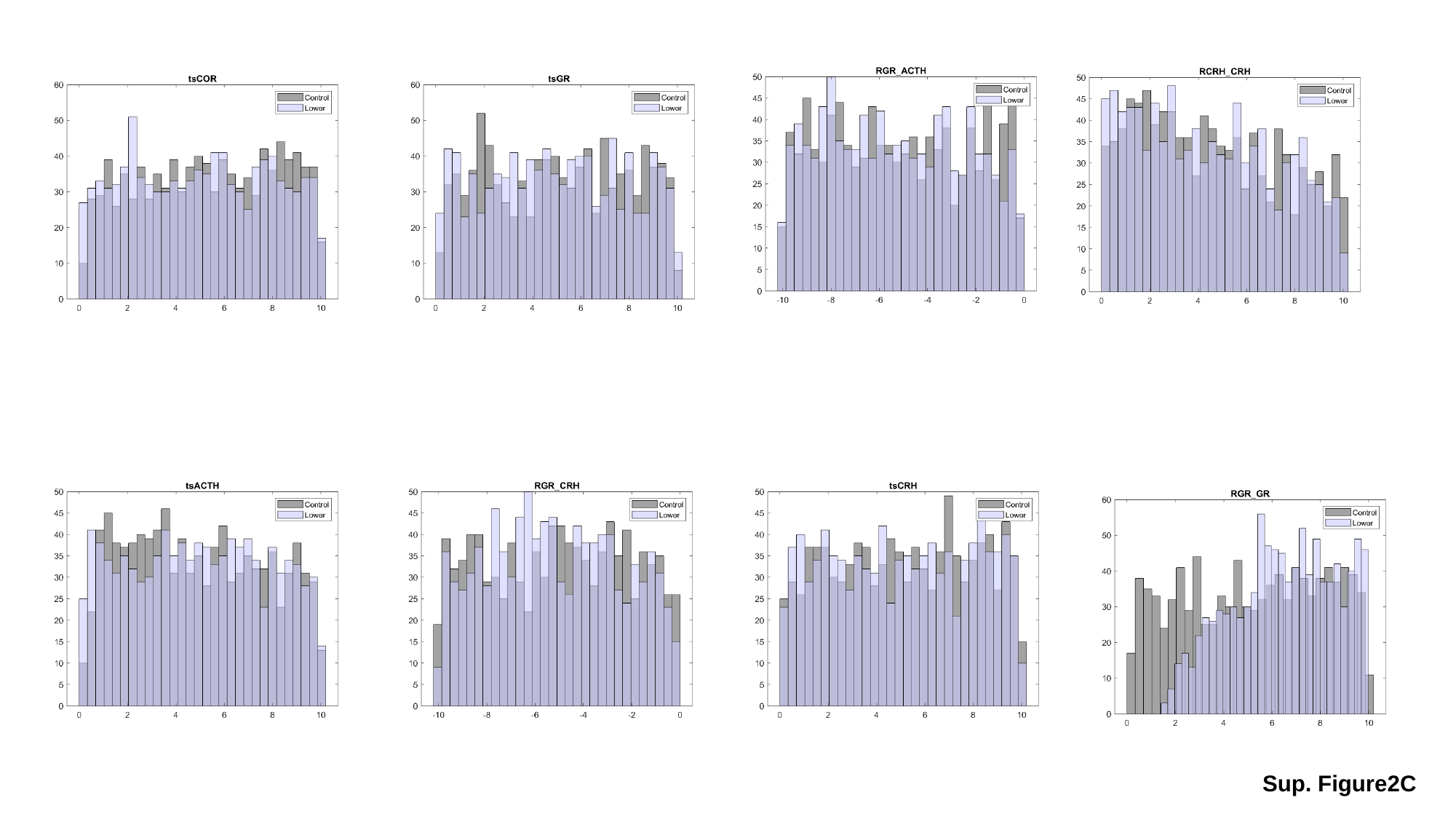

Sup. Figure2C

## Slide 5
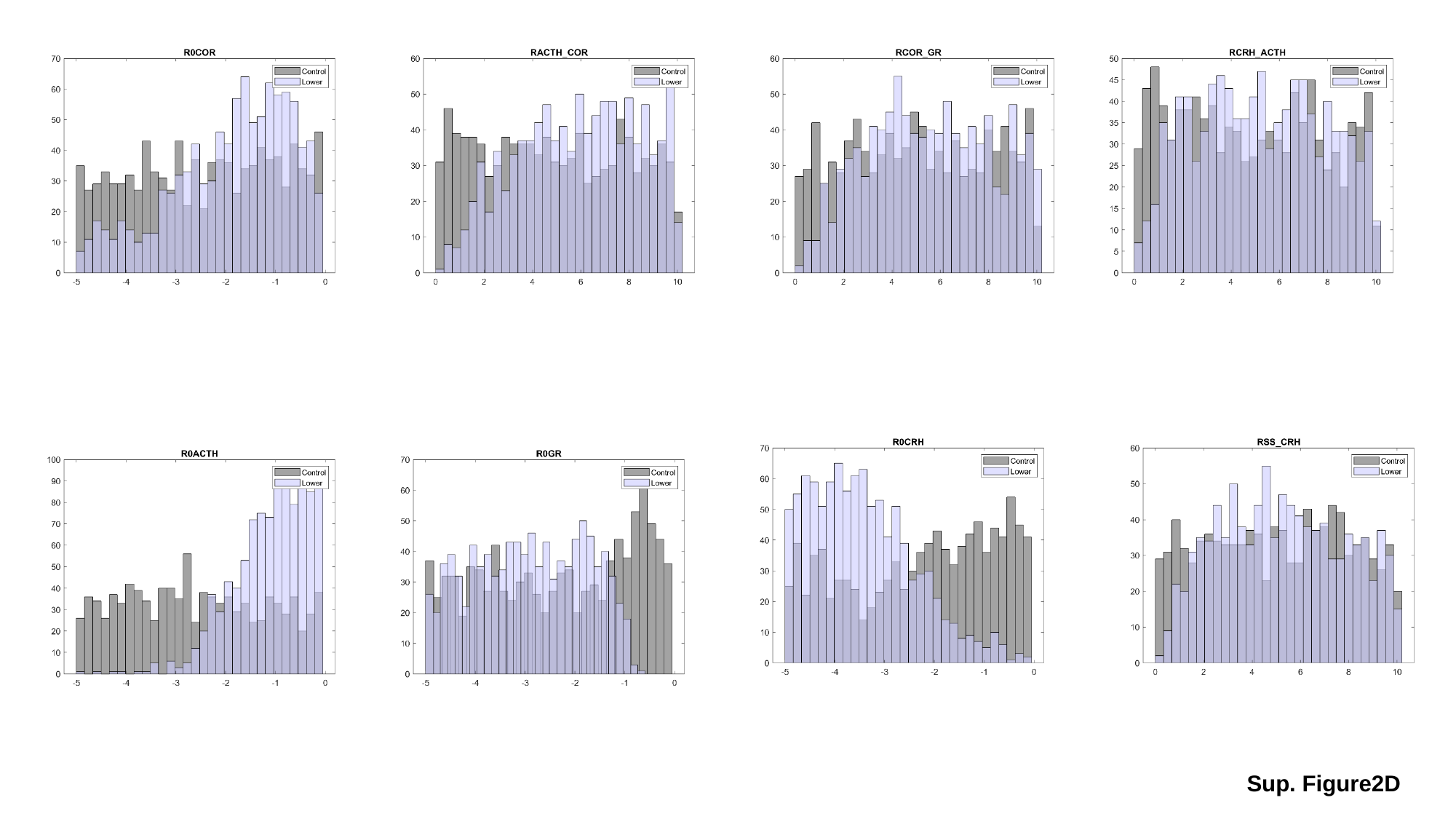

Sup. Figure2D

## Slide 6
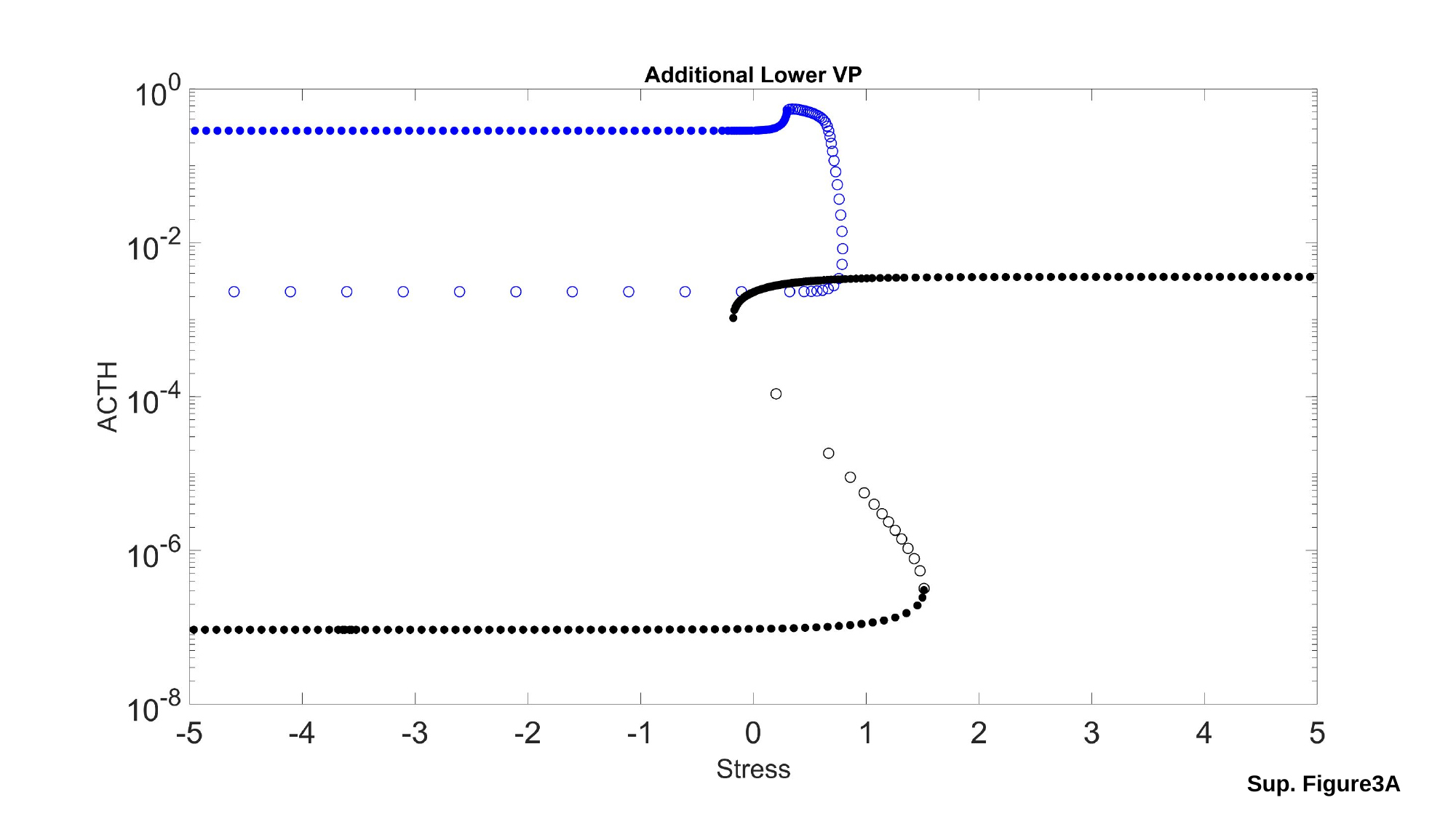

Sup. Figure3A

## Slide 7
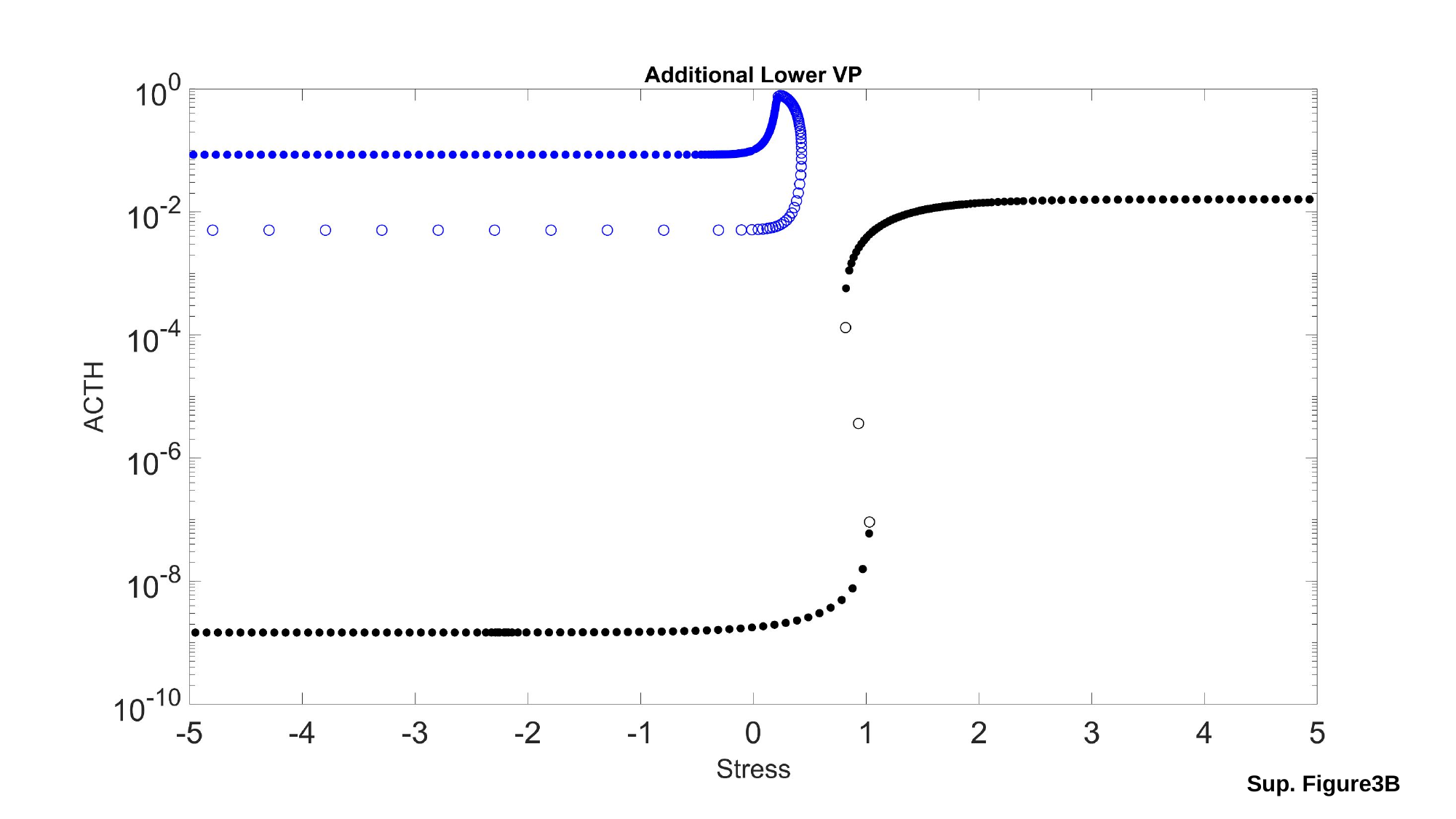

Sup. Figure3B

## Slide 8
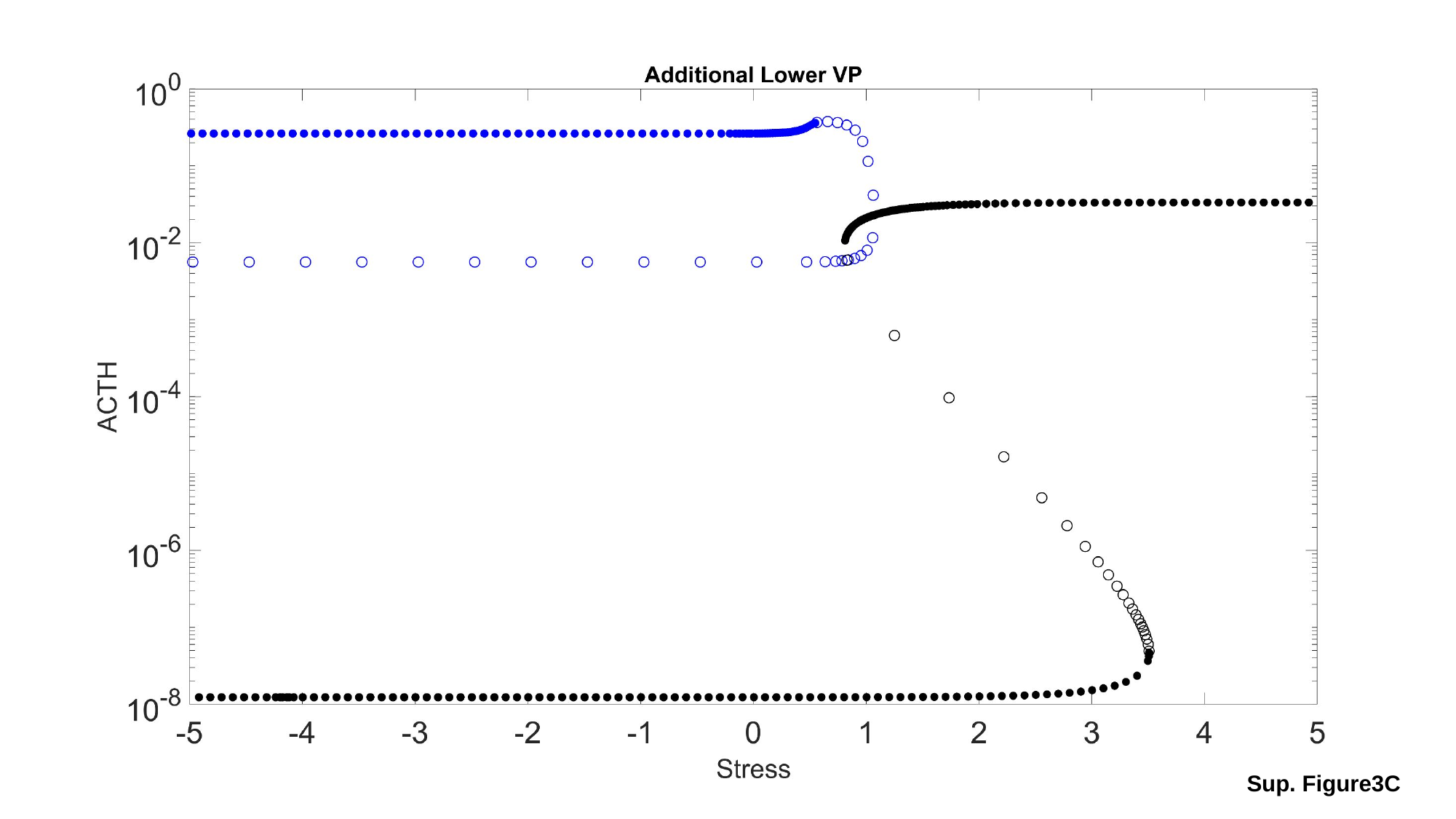

Sup. Figure3C

## Slide 9
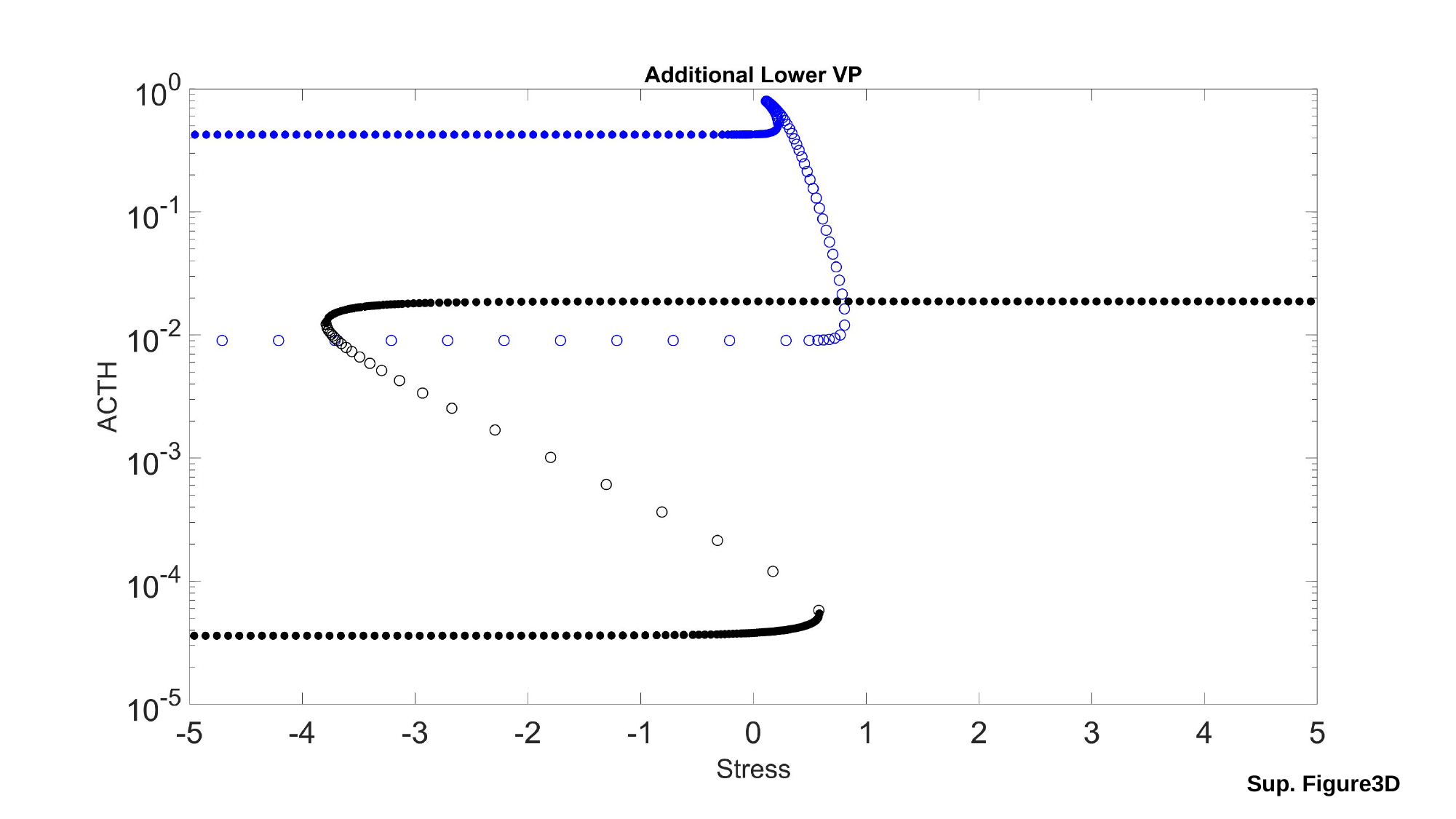

Sup. Figure3D

## Slide 10
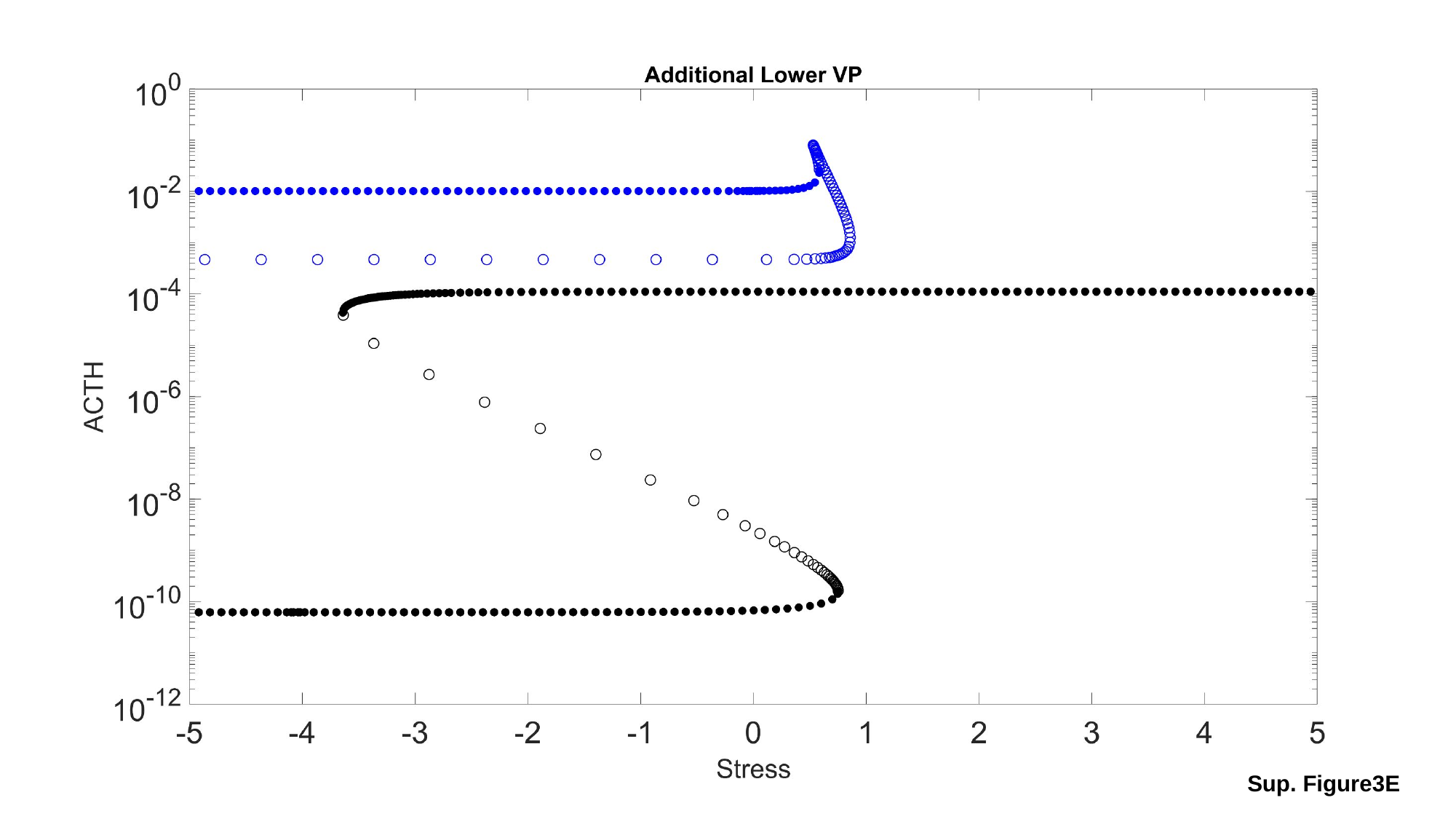

Sup. Figure3E

## Slide 11
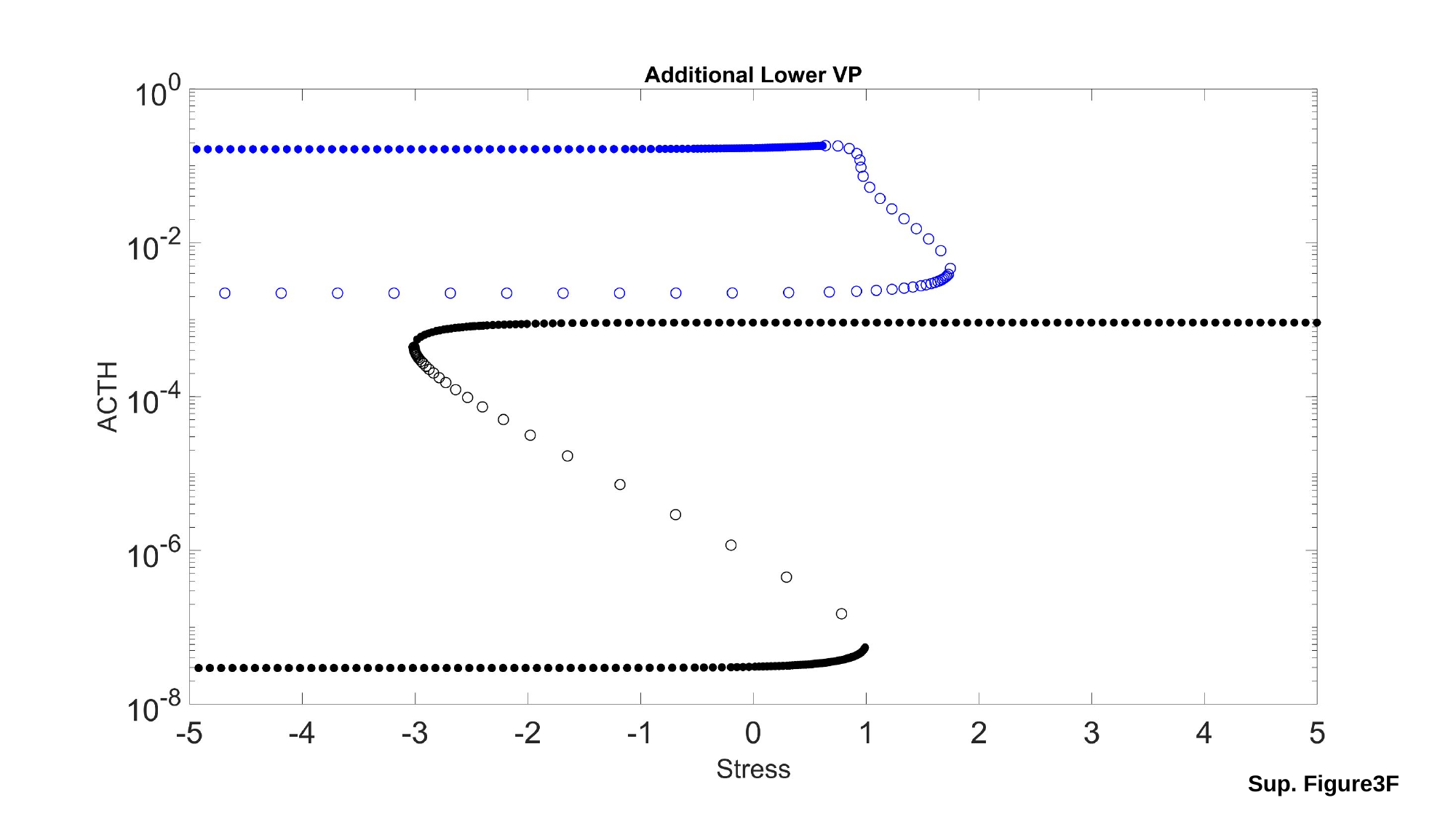

Sup. Figure3F
